# Genomic surveillance of carbapenem-resistant *Klebsiella pneumoniae* in the Republic of Moldova

**DOI:** 10.3389/fmicb.2026.1791267

**Published:** 2026-03-18

**Authors:** Svetlana Colac, Livia Tapu, Maria Anton, Diana Perde, Pimlapas Leekitcharoenphon, Saria Otani, Frank M. Aarestrup, Olga Burduniuc

**Affiliations:** 1 National Agency for Public Health, Chişinău, Moldova; 2 Nicolae Testemitanu State University of Medicine and Pharmacy, Chişinău, Moldova; 3 Research Group for Genomic Epidemiology, Technical University of Denmark, Lyngby, Denmark

**Keywords:** antimicrobial resistance mechanisms, carbapenem resistance, genomic surveillance, *Klebsiella pneumoniae*, whole-genome sequencing

## Abstract

Antimicrobial-resistant *Klebsiella pneumoniae* poses a global threat to human, animal, and environmental health. The challenges of AMR surveillance have led to the development of advanced molecular biology techniques, such as genomic sequencing, to identify the resistance gene diversity and the factors contributing to the AMR emergence and spreading. In this study, a total of 99 *Klebsiella pneumoniae* strains suspected of producing ESBL or carbapenemases were collected between January 2020 and December 2023 from nine hospital laboratories, predominantly from hospital-associated infections/isolates. The strains were analyzed using conventional methods as well as whole-genome sequencing. Phenotypic testing indicated very high resistance to key antibiotic classes, including third-generation cephalosporins (98%), fluoroquinolones (97%), aminoglycosides (95%), and carbapenems (68%). Subsequent bioinformatic analysis was performed using tools such as iTOL, the Center for Genomic Epidemiology platform, and Kleborate. Sixteen distinct MLST types were identified among the studied strains. The most common were ST395 (*N* = 56), ST377 (*N* = 12), and ST23 (*N* = 5). Phylogenetic inference revealed two major ST395 clusters spanning multiple hospitals, consistent with inter-hospital dissemination. All ST395 strains carried the resistance genes *bla*OXA-48, *bla*CTX-M-15, *bla*OXA-1, *bla*NDM-1, and *bla*TEM-1B. Bioinformatic analysis of the sequences showed that most isolates contained 7 of the 8 Col group plasmid/replicon types and 5 of the 21 Inc group types. A high virulence score was observed in 37 strains, while 2 strains showed a very high score. This includes globally transmitted strains such as ST395 and ST23 (hypervirulent), as well as the regionally dominant ST377 lineage, which was highly resistant but not hypervirulent. Our study suggests several independent introductions of *K. pneumoniae* strains, including carbapenem resistant and hypervirulent strains into Moldova, followed by spread across several hospitals. This includes globally transmitted strains such as ST395 and ST23, as well as regionally dominant strains like ST377. Comprehensive genomic surveillance of AMR has offered crucial insights for tracking multidrug-resistant *Klebsiella pneumoniae* strains. These findings help shape surveillance priorities and underline the importance of whole-genome sequencing in both research and ongoing monitoring. Such efforts are essential for guiding effective prevention and control strategies to limit the spread of resistant *K. pneumoniae* in the Republic of Moldova.

## 1 Introduction

*Klebsiella pneumoniae* is a member of the *Enterobacteriaceae* family and commonly found in the human gut microbiota (Al Balushi et al., 2022). It is the most clinically relevant *Klebsiella* species, and ranks among the top five main causative agents of antimicrobial resistant (AMR) nosocomial infections worldwide (Al-Muzahmi et al., 2023; Bortolaia et al., 2020; Carattoli et al., 2014). *K. pneumoniae* can cause a variety of infections in humans, including urinary tract infections (UTIs), respiratory tract infections (RTIs), liver abscesses, meningitis, bloodstream infections (BSI), and medical device-associated infections (Chatzidimitriou et al., 2024).

Clinically, there are two main types of *Klebsiella pneumoniae* distinguished by their virulence; broadly categorized as classical (cKp) or hypervirulent (hvKp). cKp strains are most often isolated from immunocompromised patients, typically after hospitalization, and are associated with low virulence. In contrast, hvKp strains are highly invasive, frequently cause community-acquired infections, and harbor multiple virulence factors, including a protective capsule, adhesins, toxins, and the ability to metabolize allantoin (Chatzidimitriou et al., 2024).

*K. pneumoniae* can harbor a wide range of genetic determinants encoding AMR or increased virulence. Asymptomatic carriers may shed the bacteria and act as reservoirs, promoting its transmission within healthcare settings. Inadequate or delayed antimicrobial treatment in patients with hospital-acquired bloodstream infections can lead to more severe disease and higher mortality rates within hospital units. Prompt and effective therapy, even before susceptibility results are available, is therefore essential. Thus, a solid understanding of local and regional resistance patterns is critical to guiding empiric treatment decisions.

In recent years, a number of hypervirulent *K. pneumoniae* such as ST395 (Chen C. et al., 2024; Chen T. et al., 2024) and ST23 (Choby et al., 2020), have spread globally. These isolates are also often resistant toward carbapenems making treatment options limited. There is, however, no knowledge on the potential occurrence in Moldova.

Understanding whether infections with *K. pneumoniae* are a result of transmission between patients or unrelated events is critical for implementing control measures. Transmission between patients typically calls for strengthened hygienic and transmission prevention, while isolated cases often require adjustments to individual treatment strategies (David et al., 2023).

Studying the genetic diversity of *K. pneumoniae* is essential for identifying its main resistance mechanisms and developing evidence-based recommendations for rational antibiotic therapy. Phylogenetic analyses of the circulating strains can provide information on emergence of novel clones and the extent of patient-to-patient transmission, and thereby guiding the formulation of effective infection control strategies.

Our study investigated 99 ESBL or carbapenem resistant *K. pneumoniae* strains isolated between 2020 and 2023 across Moldova using whole genome sequencing.

## 2 Materials and methods

### 2.1 Bacterial strains

Between January 2020 and December 2023, a total of 99 *K. pneumoniae* isolates, suspected to be either ESBL-producing or resistant to carbapenems—were collected through the network of laboratories within the National System for Epidemiological Surveillance of Antimicrobial Resistance (SNSE RAM). SNSE RAM is coordinated by the National Agency for Public Health (an administrative authority under the Ministry of Health) and operates as part of routine, passive public health surveillance aligned with Central Asian and Eastern European Surveillance of Antimicrobial Resistance (CAESAR) and Global Antimicrobial Resistance and Use Surveillance System (GLASS) requirements. The national AMR surveillance system was established by the Ministry of Health decision No. 711 (7 June 2018) and replaced by Order of the Ministry of Health No. 407/2025. Isolates originate from a nationwide laboratory network (regional public health centers and public/private medical institutions), and de-identified strain registration forms are forwarded weekly to the National Reference Laboratory for centralized entry and reporting. In each participating laboratory, isolates were cultured on MacConkey agar and chromogenic UTI agar. Strain identification was carried out via the automated VITEK^®^ 2 COMPACT system (bioMérieux, France), and antimicrobial susceptibility testing was performed using both the disk diffusion method and the VITEK 2 system. All isolates identified as *K. pneumoniae* and resistant to either cephalosporins or carbapenems were preserved on sterile cryovials (CryoInstant, Deltalab) at −80 °C for further analysis (Supplementary Table 1). The strains were isolated from CSF—3.7% (*n* = 3), blood—44.4% (*n* = 44), and urine—52.52% (*n* = 52).

### 2.2 Susceptibility to antimicrobial agents

Antimicrobial susceptibility testing was initially performed within individual laboratories using the Vitek 2C automated system (bioMèrieux, France). Upon arrival at the National Agency for Public Health, the testing was repeated. For isolates identified as pan-resistant to colistin by the Vitek 2C panel, additional testing was conducted. Until 2023, colistin susceptibility was assessed using commercial MIC-Strip Colistin kits (MERLIN Diagnostika GmbH, Germany); from 2023 onward, testing was performed using Sensititre (Thermo Scientific™ Sensititre™ Complete Automated AST System). All methods used a pure 18–24 h culture suspended in normal saline and adjusted to a McFarland standard of 0.5 (approximately 1–2 × 10^8^ CFU/mL), using the Densichek device (bioMèrieux, France), in accordance with the manufacturer’s instructions. Within 15 min, the suspension was spread onto the surface of Mueller-Hinton Agar (HiMedia, India) and left at room temperature for several minutes to allow absorption. Antibiotic disks (MAST Group Ltd., UK) were then placed on the inoculated plates using sterile tweezers within 15 min of application. Plates were incubated at 37 °C for 18–24 h. Antibiotics were selected according to the EUCAST standard (version corresponding to the year of sampling and strain testing) as follows: ampicillin–clavulanic acid (AMC 10/20 μg), piperacillin/tazobactam (TZP 30/6 μg), ceftriaxone (CRO 30 μg), ceftazidime (CAZ 10 μg), cefotaxime (CTX 5 μg), cefoxitin (FOX 30 μg), (IPM 10 μg), meropenem (MEM 10 μg), ertapenem (ETP 10 μg), amikacin (AMK 30 μg), gentamicin (GEN 10 μg), tobramycin (TOB 10 μg) and ciprofloxacin (CIP 5 μg), ofloxacin (OFX 5 μg), levofloxacin (LVX 5 μg). *E. coli* (ATCC 25922) was used as a sensitive control strain. Colistin MIC testing was performed selectively (upon request or in isolates showing near-total resistance/pan-resistance on routine panels), consequently, colistin results are not available for all isolates and years. MDR was reported using the CAESAR combined resistance indicator for *Klebsiella pneumoniae* (combined resistance to fluoroquinolones, third-generation cephalosporins, and aminoglycosides) and was calculated only where this indicator was applicable and reported.

### 2.3 DNA extraction, whole genome sequencing and result analysis

Bacterial DNA extraction was performed using the Qiagen DNeasy Blood & Tissue kits according to the manufacturer’s protocol (Illumina, Inc., San Diego, CA, United States). Whole genome sequencing (WGS) of the isolates was performed using Illumina paired-end sequencing on the NovaSeq 6000 similar to Davoudabadi et al. (2021). Raw paired-end reads were submitted to ENA under project number PRJEB90294. Accession number for each samples including metadata can be found in Supplementary Table 1.

Bioinformatic analysis was carried out using the services provided by the Center for Genomic Epidemiology.^1^ The ResFinder tool (version 4.6.0) (De Oliveira et al., 2020) was used to analyze sequencing data for identifying antimicrobial resistance (AMR) genes, including the specific genomic positions of point mutations associated with AMR. PlasmidFinder (version 2.1) (Effah et al., 2020) was used to detect plasmids. Multilocus Sequence Typing (MLST) was determined using MLST version 2.0 (European Centre for Disease Prevention and Control, 2021), and SNP phylogenetic trees were constructed using CSI Phylogeny 1.4 (European Centre for Disease Prevention and Control, 2024), with *K. pneumoniae* ST395 as the reference genome. The resulting phylogenetic tree was visualized and managed using the iTOL online platform.^2^ To identify hypervirulent *K. pneumoniae* (HvKp), the Kleborate tool provided by PathogenWatch^3^ was used (García-González et al., 2025).

## 3 Results

### 3.1 Origin of the *Klebsiella pneumoniae* strains

Of the 99 *K. pneumoniae* strains, 96 (96.96%) were isolated from patients with hospital-acquired infections, of which 35 (35.35%) came from adult intensive care units, two from pediatric intensive (2.02%) care and 59 (59.59%) from other departments (Table 1 and Supplementary Figure 1). The remaining three isolates (3.03%) were of community origin, detected in outpatients. The “other departments” included internal medicine (6.06%, *n* = 6), pediatrics (1.01%, *n* = 1), urology (6.06%, *n* = 6), surgery (2.02%, *n* = 2), and various other departments (44.44%, *n* = 44) (Supplementary Figure 1). The proportion of male patients was 46.46% (*n* = 46) and female patients 53.53% (*n* = 53), with a male-to-female ratio of 0.87 (see Table 1). Most patients were over 65 years of age, with only a few in younger age groups (Table 1). Of the 99 *K. pneumoniae* isolates, 44 (44.4%) were obtained from blood, 52 (52.5%) from urine, and 3 (3.0%) from cerebrospinal fluid.

**TABLE 1:** Demographic profile of patients with *Klebsiella pneumoniae* isolates.

Age groups	< 1 year and 1 year inclusive		18–35 years		36–50 years		51–65 years		> 65 years		Total
	*n*	%	*n*	%	*n*	%	*n*	%	*n*	%	
Male	5	10.87	1	2.17	5	10.87	14	30.44	21	45.65	**46**
Female	0	0.0	11.32	16.98	9	16.98	9	16.98	29	54.72	**53**
											**99**
**Age groups**	**< 1 year and 1 year inclusive**		**18–35 years**		**36–50 years**		**51–65 years**		**> 65 years**		**Total**
	***n***	**%**	***n***	**%**	***n***	**%**	***n***	**%**	***n***	**%**	
Intensive care unit	2	4.35	2	4.35	4	8.69	11	23.92	27	58.69	**46**
Other departments	3	6.0	5	10.0	9	18.0	11	22.0	22	44.0	**50**
Ambulatory care	0	–	0	–	1	33.33	1	33.33	1	33.33	**3**
											**99**

Bold values indicate the total number of patients.

### 3.2 Antimicrobial susceptibility testing

Resistance to third-generation cephalosporins was confirmed in 98% of the strains (Table 2). Additionally, high prevalence of resistance to aminoglycosides (95%), and fluoroquinolones (97%) were found. Resistance to carbapenems was identified in 68% of the strains. Colistin susceptibility results were available for 42 isolates of which five (12%) were resistant.

**TABLE 2:** Antimicrobial resistance among 99 *Klebsiella pneumoniae* strains isolated from nine hospitals across Moldova.

The agent or group of antimicrobial agents tested	No. of tested strains	No. of non-susceptible strains (*R*)	%	No. of susceptible strains (S)	%
Resistance to antimicrobial agents					
Amoxicillin-clavulanic acid	99	94	94.95	5	5.05
Piperacillin tazobactam	99	91	91.92	8	8.08
Cefotaxime	98	96	97.96	2	2.04
Ceftriaxone	94	92	97.87	2	2.13
Ceftazidime	99	95	95.96	4	4.04
Ertapenem	99	84	84.85	15	15.15
Imipenem	99	57	57.58	42	42.42
Meropenem	99	65	65.66	34	34.34
Amikacin	99	66	66.67	33	33.33
Gentamicin	99	86	86.87	13	13.13
Tobramycin	99	94	94.95	5	5.05
Ciprofloxacin	99	96	96.97	3	3.03
Levofloxacin	99	90	90.91	9	9.09
Ofloxacin	90	88	97.78	2	2.22
Resistance to groups of antimicrobial agents					
Cephalosporins IIIrd generation (cefotaxime/ceftriaxone/ceftazidime)	99	97	97.98	2	2.02
Carbapenems (imipenem/meropenem)	99	67	67.68	32	32.32
Aminoglycosides (gentamicin/tobramycin)	99	94	94.95	5	5.05
Fluoroquinolones (ciprofloxacin/levofloxacin/ofloxacin)	99	96	96.97	3	3.03
Multidrug-resistance^a^	99	51	51.52	NA^b^	NA^b^

^a^Multidrug-resistance is here defined as combined resistance to at least one antibiotic from each of three groups: fluoroquinolones (ciprofloxacin, levofloxacin and/or ofloxacin), III-generation cephalosporins (cefotaxime, ceftriaxone and/or ceftazidime) and aminoglycosides (gentamicin and/or tobramycin). Isolates lacking data for any of these groups are excluded from the multidrug resistance analysis. ^b^Not applicable (i.e., the category was not tested/reported according to EUCAST and/or CAESAR).

### 3.3 Confirmation of ESBL and carbapenemase production

**FIGURE 1:**
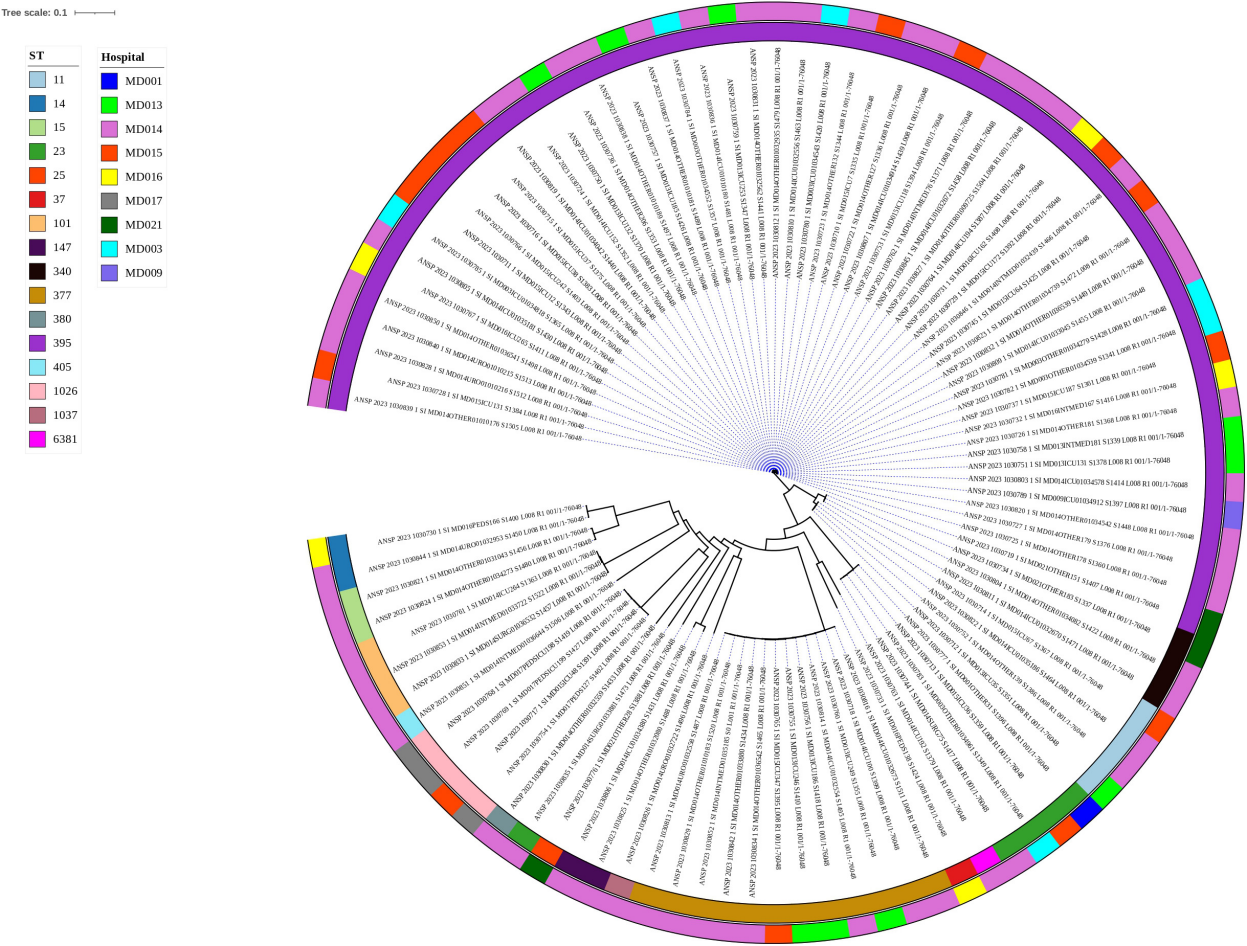
Phylogenetic tree of ST395 isolates inferred from core genome SNP analysis by CSI phylogeny and visualized with iTOL.

ESBL production was further confirmed using the double disk synergy assay and the combined disk assay (MastDisk Combi). Screening for carbapenemase production was carried out with the CARBA PaCE colorimetric test (MAST Group Ltd., UK). Among the tested strains, 22 (22.22%) were ESBL producers, 71 (71.71%) produced carbapenemases, and 2 (2.02%) produced both enzymes simultaneously (Figure 1). The presence of genetic determinants of carbapenemases (blaKPC, blaOXA-48, blaNDM, blaVIM, blaIMP) was confirmed by the commercial real-time PCR kits AmpliSens^®^ MDR KPC/OXA-48-FL and AmpliSens^®^ MDR MBL-FL, following the manufacturer’s instructions (Amplisens, Russia).

### 3.4 Multi-locus sequence typing

A total of 16 different MLST types were found among the strains. The most common types were ST395 (*N* = 56), ST377 (*N* = 12) and ST23 (*N* = 5) (Table 3). ST395 was found in most of the hospital institutions, including from primary (MD009A), secondary (MD003A, MD013A, MD016A) and tertiary (MD014A, MD015A, MD021A) hospitals and outpatient institution (Table 3 and Supplementary Tables 4.1, 4.2).

**TABLE 3:** MLST types found among the different hospitals.

ST	Hospitals											Total
	Primary	Secondary			Tertiary						Ambulatory	
	MD009A	MD003A	MD013A	MD016A	MD014A	MD015A	MD015B	MD017A	MD021A	MD21D (private)		
11			1		2		1					4
14				1	1							2
15					2							2
23		1			2		1				1	5
25						1						1
37				1								1
101					3							3
147					2							2
340					2					1		3
377			3		8	1						12
380					1							1
395	1	5	5	3	30	9			1		2	56
405					1							1
1,026						1		3				4
1,037					1							1
6,381						1						1
Total	1	6	9	5	55	13	2	3	1	1	3	99

### 3.5 Phylogenetic analysis

**FIGURE 2:**
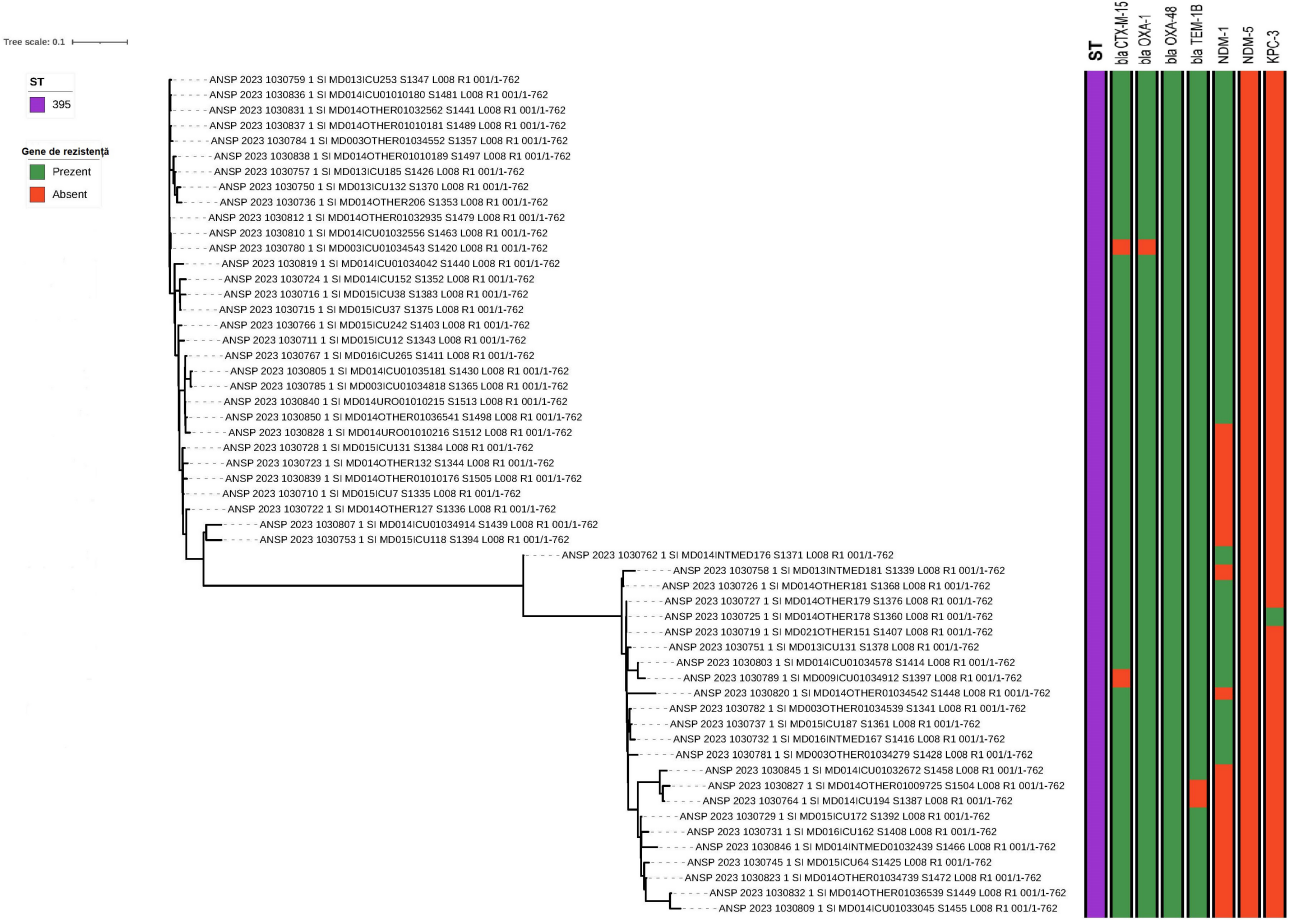
SNP-based Phylogenetic tree of 99 sequenced genomes of *Klebsiella pneumoniae* from nine different hospitals across Moldova.

Strains within the same group (ST) were genetically more similar to each other than to isolates from different STs (Figure 2). Generally, a high genetic heterogeneity was observed among the sample, but several clusters of almost identical strains were found, which may indicate an epidemiological link between some of the samples studied. Notably, isolates of ST395 and ST377 appeared in several different hospitals.

The 56 isolates belonging to ST395 were further investigated by a separate phylogenetic tree, revealing two different clusters; cluster-1 with 31 strains and cluster-2 with 24 strains, as well as a single non-cluster-related strain (Supplementary Figure 2). The number of SNPs between the isolates of each cluster ranged from 0 to 72 and 9 to 99, in cluster 1 and 2, respectively, and closely related strains were detected at different hospitals. This suggest both circulation between different hospitals, as well as possibly multiple introductions into Moldova from other countries. More details are provided in Supplementary material.

**FIGURE 3:**
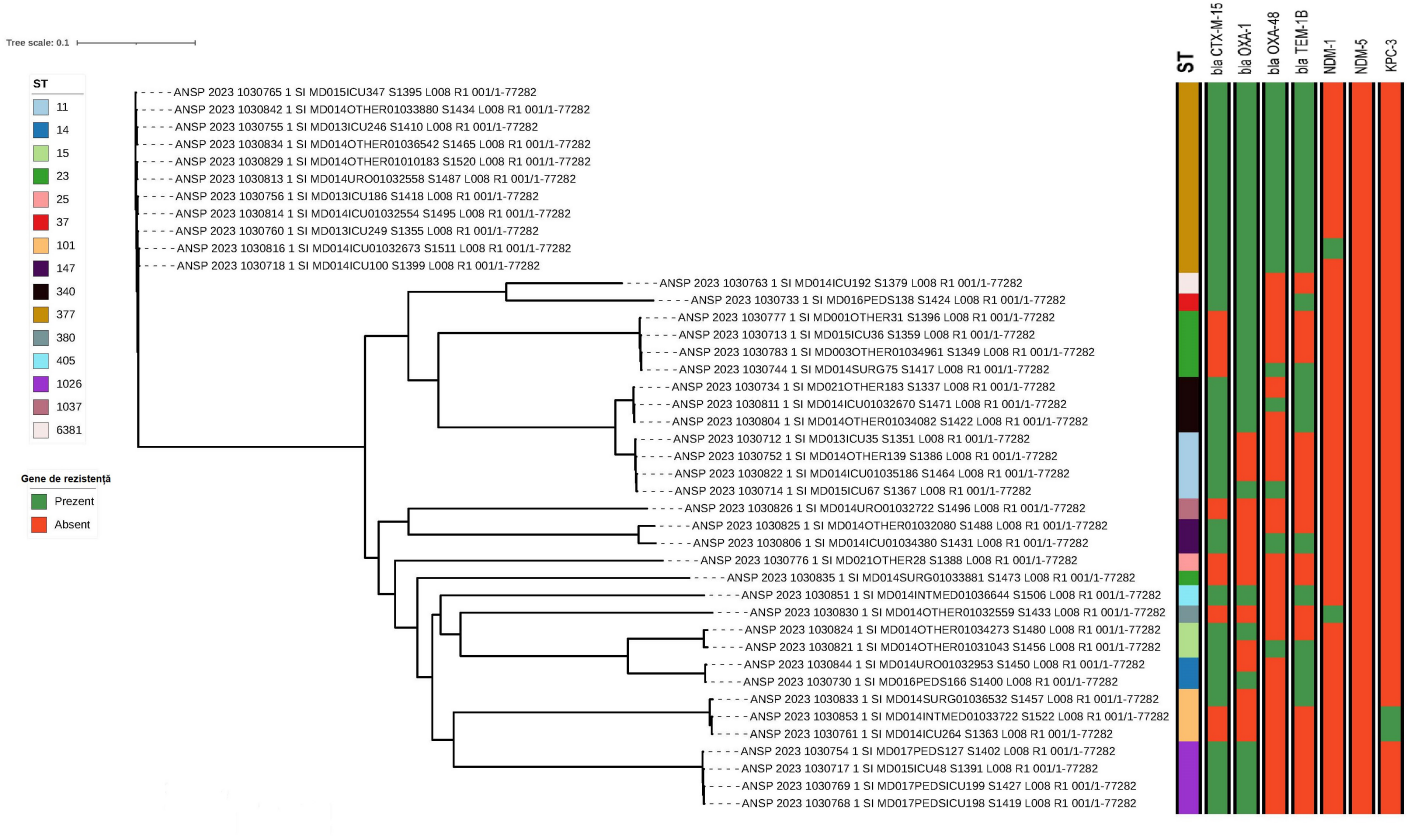
Phylogenetic tree of isolates of ST-types other than 395 inferred from core genome SNP analysis. Selected antimicrobial resistance genes of relevance for *Klebsiella pneumoniae* were also included in the figure.

Beyond ST395, the phylogeny of non-ST395 isolates (Figure 3) revealed a heterogeneous population structure comprising multiple sequence types, including ST377, ST23, ST11, ST14/ST15 and several less frequent STs. Within the most frequent non-ST395 lineages (ST377 and ST23), isolates formed compact sub-clusters with short branch lengths, consistent with recent shared ancestry. These lineage-specific clusters were detected across the participating hospitals (Table 3), suggesting circulation beyond a single facility.

### 3.6 Hypervirulent *Klebsiella pneumoniae*

Using the PathogenWatch tool (Kleborate) we identified HvKp isolates of several ST types and clonal lineages lines (see Supplementary Table 5). HvKp are typically more virulent than classical *K. pneumoniae* (cKp) and are known to cause community infections, especially in healthy individuals, spreading both within healthcare facilities and in the wider community. HvKP strains, especially multidrug-resistant hvKP clones (MDR-hvKP), represent a serious threat to public health (Greičius et al., 2024; Hallal Ferreira Raro et al., 2023; Ibik et al., 2023).

Virulence information has been summarized using a score from 0 to 5, reflecting the overall hierarchy of virulence and associated genetic loci identified in the literature over the past two decades.

We found 37 strains with a high virulence score of 4 and two strains with the highest score of 5. The two strains scoring 5 belonged to the ST23 K1 and ST380 K2 clonal lineages. Of the strains scoring 4, 33 were ST395 K39, three were ST23 K57, and one was ST25 K39. The classification of these *K. pneumoniae* strains as highly hypervirulent is alarming, especially given the global spread of hvKp lineages like ST23 and ST395, which represent a significant worldwide threat (Kaas et al., 2014; Kovács et al., 2017; Lam et al., 2021).

### 3.7 Analysis of antimicrobial resistance genes in *Klebsiella pneumoniae*

The sequenced *K. pneumoniae* genomes were examined for antimicrobial resistance genes (ARGs), including those responsible for extended-spectrum beta-lactamases and carbapenemases. A complete list of all ARGs is given in Supplementary Table 3. Some of the clinically most relevant ARGs are depicted in Figure 1 for strains of ST395 and Figure 3 for non-ST395.

All ST395 strains carried the *bla*OXA-48 gene, which confers resistance to carbapenems, and the vast majority also had the *bla*CTX-M-15, *bla*OXA-1, *bla*NDM-1, and *bla*TEM-1B genes. Only one strain carried the *bla*KPC-3 gene.

More variation in the presence of beta-lactamase genes was observed in the non-ST395 isolates and with the presence or absence of genes not necessarily following the phylogeny. No genes encoding cephalosporin resistance were observed in strains 1030826, 1030776 or 1030835.

Beyond the resistance genes shown in Figure 1, 3, many other antimicrobial resistance genes were found among the isolates with most isolates harboring aac(6′)-Ib-cr (93 isolates), blaOXA-1 (88 isolates), different blaSHV-genes (all isolates), blaTEM-1B (79 isolates), blaCTX-M-15 (86 isolates), OqxA and OqxB (98 isolates), and sul1 (83 isolates), qnrS1 (74 isolates), while a more limited number of isolates harbored fosA (98), fosA6 (12), catA1 (69), tet(A) (69), dfrA1 (74), qacE (64), ant(2″)-Ia (28), and ant(3″)-Ia (28). 16S rRNA Methyltransferases amrA and rmtC encoding high-level resistance to aminoglycosides were observed in 16 and 39 isolates, respectively. Notably, armA was found in all 12 ST377 isolates, but also found in four isolates of other STs. rmtC was primarily observed in ST395 isolates, being present in 37 (66%) of the isolates, but also found in a few other STs as well.

### 3.8 Analysis of plasmids in *Klebsiella pneumoniae*

Most isolates harbored 8 types of plasmids/replicons from the Col/group, including ColRNAI, Col440II, Col156, Col (MG828), Col (BS512), ColpVC, Col440I, and Col(pHAD28). The predominant plasmids were ColRNAI (72 isolates), Col440II (87 isolates), Col156 (53 isolates), while Col (MG828) was found only in 9 strains (Supplementary Figure 3). These Col plasmids showed alignment coverage between 92% and 100%, with sequence similarity ranging from 99% to 100%. Following the analysis, 25 multiple replicon plasmids were detected, including IncFIA, IncFIB, IncFII, IncR, IncM2, IncHI1B, IncX1, IncX3, IncQ1, IncB/O/K/Z, IncHI2, IncHI2A, IncI1-I, IncY, IncL (Supplementary Figure 3). Notably, five strains (1030840, 1030844, 1030736, 1030777, 1030732) that were isolated at different times from two hospitals and one outpatient facility and representing different sequence types (ST–395, 14, 23), all carried repB plasmids.

## 4 Discussion

A total of 99 strains of *K. pneumoniae* suspected to be ESBL or carbapenem producers were isolated from blood and urine during 2020–2023 by the Moldavian National Antimicrobial Resistance Epidemiological Surveillance System.^4^ The isolates were sequenced and analyzed, confirming the suspected resistance phenotypes in most cases and thus validating the quality of the current clinical microbial diagnostic at the individual hospitals. The isolates were obtained from 11 different centers, but since the number of isolates provided from each center differs and we only included isolates suspected to be ESBL and/or carbapenem producers this could have included a bias and do not reflect the overall prevalence of *K. pneumoniae* in Moldova. Most isolates tested resistant to multiple antimicrobial agents, showing the potential difficulty in treating these strains.

Phylogenetic analyses showed a broad diversity of *K. pneumoniae* in Moldova and also presence of multiple also closely related types at different hospitals. Thus, suggesting multiple independent introductions of ESBL- and carbapenemase-producing strains into Moldovan hospitals.

The most commonly identified type was ST395. All ST395 isolates carried the *bla*OXA-48 gene, and many were classified as hypervirulent. First reported in a 2010 outbreak in France, ST395 has since spread globally (Chen C. et al., 2024). We here report the first known isolate identified in Moldova in 2020, although it cannot be excluded that the strains had been circulating earlier. The ST395 strains in Moldova grouped into two clusters, which aligns with the two main clusters found in global data (Chen C. et al., 2024; Larsen et al., 2012; Leekitcharoenphon et al., 2021), despite showing less diversity in our findings. The frequent co-occurrence of *bla*OXA-48, *bla*CTX-M-15, and high virulence scores in ST395 strains supports the ongoing convergence of resistance and virulence traits in epidemic *K. pneumoniae* clones (García-González et al., 2025).

ST377 was the second most common type observed. This type has only been rarely reported and to the best of our knowledge only from Iran, Russia and Turkey (Marr and Russo, 2019; Munoz-Price et al., 2013; Qi et al., 2011). These findings may indicate geographical dissemination, although further studies are required to confirm the extent and patterns of spread. All 12 isolates consistently harbored *bla*CTX-M-15, *bla*OXA-1, *bla*OXA-48, and *bla*TEM-1b, with one isolate also carrying *bla*NDM-1. The strains showed a high degree of genetic similarity and were identified across four different hospital units, suggesting recent transmission events. Although the high prevalence of resistance is concerning, the strains were not classified as hypervirulent. Nonetheless, the ongoing transmission in Moldova—and potentially in neighboring countries— should be a focus for future surveillance efforts.

In addition, we identified hypervirulent ST23 isolates, a clone that has recently emerged in several European countries (Russo and Marr, 2019). All four ST23 strains carried *bla*CTX-M-55 and *bla*SHV-51.

Four isolates were identified as ST11, a sequence type previously reported as a predominant clone among carbapenem-resistant *Klebsiella pneumoniae* isolates in Asia (Russo and Marr, 2019), often associated with the *bla*KPC-1 gene (Russo et al., 2018). None of the isolates in our collection harbored *bla*KPC-1, but one isolate contained blaOXA-48 and they all had *bla*CTX-M-15 as well. ST11 is also a major clone on a global scale (Shaidullina et al., 2023), and carbapenem-resistant isolates have been frequently reported in the Balkans (Shelenkov et al., 2020).

A genomic surveillance study on ST307 conducted in Wales (UK) analyzed 540 clinical, environmental, and screening isolates collected between 2007 and 2020. It identified the ST307 clone as a high-risk strain that spread both between and within hospitals, with undetected persistence for many years prior to an acute outbreak (Skachkova et al., 2019).

A study conducted in Spain between 2017 and 2019 analyzed 1,768 *Klebsiella pneumoniae* isolates collected from eight hospitals and revealed substantial genetic diversity. Among these were globally recognized high-risk clones such as ST307 and ST11, carrying resistance determinants to third-generation cephalosporins and carbapenems. Notably, approximately 50% of the isolates were linked to transmission events, with 70.5% occurring within hospital settings (Spadar et al., 2022).

The clustering of very similar ST395 and ST377 strains in our study across multiple hospitals suggests sustained transmission. This underscores the need to strengthen infection prevention protocols and implement routine molecular surveillance in high-risk units.

Thirty-nine of our isolates had a virulence score of 4 or 5, which is high compared to previous studies (García-González et al., 2025), underscoring the potential public health impact of the transmission of these strains. Analysis of the plasmids in the tested isolates showed that ColRNAI, Col440II, and Col156 were the most predominant types in most cases. Plasmids carry genes that provide bacteria with useful traits, like antibiotic resistance. Additionally, the presence of repB plasmids in unrelated lineages further supports plasmid-mediated dissemination of resistance genes across *K. pneumoniae* populations in Moldova. Studies support that repB plasmids, particularly R1701, can mediate the dissemination of resistance genes across clonal lineages of *Klebsiella pneumoniae*, highlighting the importance of molecular surveillance and plasmid monitoring in preventing the spread of antimicrobial resistance (Wyres et al., 2020; Yao et al., 2024).

## 5 Conclusion

In conclusion, our study suggests several independent introductions of *K. pneumoniae* strains, including carbapenem and hypervirulent strains into Moldova, followed by their spread across several hospitals. This includes globally transmitted strains such as ST395 and ST23, as well as regionally dominant strains like ST377, as well as multiple strains with high virulence scores. The transmission of these high-risk clones highlights the urgent need to strengthen infection prevention and control measures within healthcare settings. Timely diagnosis, effective infection control, and antimicrobial stewardship are crucial for combating antimicrobial resistance, particularly in hospital settings. Supporting ongoing research and surveillance through whole-genome sequencing (WGS) is essential for identifying high-risk clones, allowing prompt infection control measures to prevent the further spread of carbapenem-resistant *Enterobacteriaceae* across the Republic of Moldova.

## Data Availability

The metagenomic sequencing data (FASTQ) have been deposited in the European Nucleotide Archive (ENA) under the project accession number PRJEB90294.

## Footnotes

1
https://www.genomicepidemiology.org/services/

2
https://itol.embl.de/

3
https://pathogen.watch/

4
https://ms.gov.md/wp-content/uploads/2025/05/Ordin-407_08052025123745.pdf
