# Supplementary material for: Genomic surveillance of carbapenem-resistant Klebsiella pneumoniae in the Republic of Moldova
Source: Front Microbiol. 2026 Mar 18;17:1791267. doi: 10.3389/fmicb.2026.1791267 (PMC13038923; doi:10.3389/fmicb.2026.1791267)
Supplement: Supplementary file 1 [file Data_Sheet_1.docx]

**SUPPLEMENTARY MATERIAL**

SUPPLEMENTARY RESULTS

**Detailed description of the phylogenetic narrative for isolates belonging to ST395**

Separate phylogenies were constructed for each cluster (Figure 2), where the number of SNPs between the isolates ranged from 0-72 and 9-99 in cluster 1 and 2, respectively. Among strains of cluster 1, the first invasive isolates appeared in 2020 in the MD015A - a republican- level hospital, specifically in the Intensive Care Unit (1030710, 1030711, 1030715, 1030716). The following year, ST395 expanded within the same hospital (sample 1030728), and closely related strains were also found at another tertiary hospital, MD014A (samples 1030722, 1030723), in both hospitals - other therapeutic departments. In 2022, additional isolates appeared again in MD015A (samples 1030753, 1030766), as well as in intensive care units and other departments within secondary-level hospitals MD013A (samples 1030750, 1030757, 1030759) and MD016A (sample 1030767). The spread continued into 2023, reaching municipal hospitals such as MD003A (samples 1030780, 1030785 from urine). Moreover, the cluster also extended to other republican institutions, such as MD014A’s Intensive Care Unit (samples 1030805, 1030810, 1030819, 1030836 - urine), as well as the urology department (samples 1030828, 1030840 – urine), and the ambulatory district at the Riscani institution (sample 1030807 – urine), showing that these strains are present not only in municipal and republican hospitals but also in outpatient facilities throughout the Republic of Moldova.

The second cluster includes 23 *Klebsiella pneumoniae* strains ST395. The first invasive isolate was identified in 2020 in a tertiary institution MD021A (1030719). Next year, additional invasive isolates appeared in several hospitals: MD014A (1030725, 1030726, 1030727 – blood, 1030832 - urine); MD015A (1030729, 1030737 – blood); and MD016A (1030731, 1030732 - blood). In 2022, strains from the second cluster were found in MD013A’s Internal Medicine Unit (1030758 - blood) and other department (1030751 - blood), as well as in the Intensive Care Units of MD014A (1030764 - blood). The number of isolates increased further in 2023, with 12 strains identified across multiple units as follows: MD003A (1030781, 1030782 - urine) and MD009A (1030879 - urine); MD014A’s Internal Medicine Department (1030846 - urine), Intensive Care Unit (1030803, 1030809, 1030845 – urine), Urology Department (1030841 - urine) and Other Departments (1030820, 1030823, 1030827 - urine); MD015A (1030745 - blood).

An invasive strain of *Klebsiella pneumoniae* ST395 was not included in any cluster, it was isolated in 2022, within the tertiary level hospital MD014A, within a therapeutic department.

SUPPLEMENTARY FIGURES


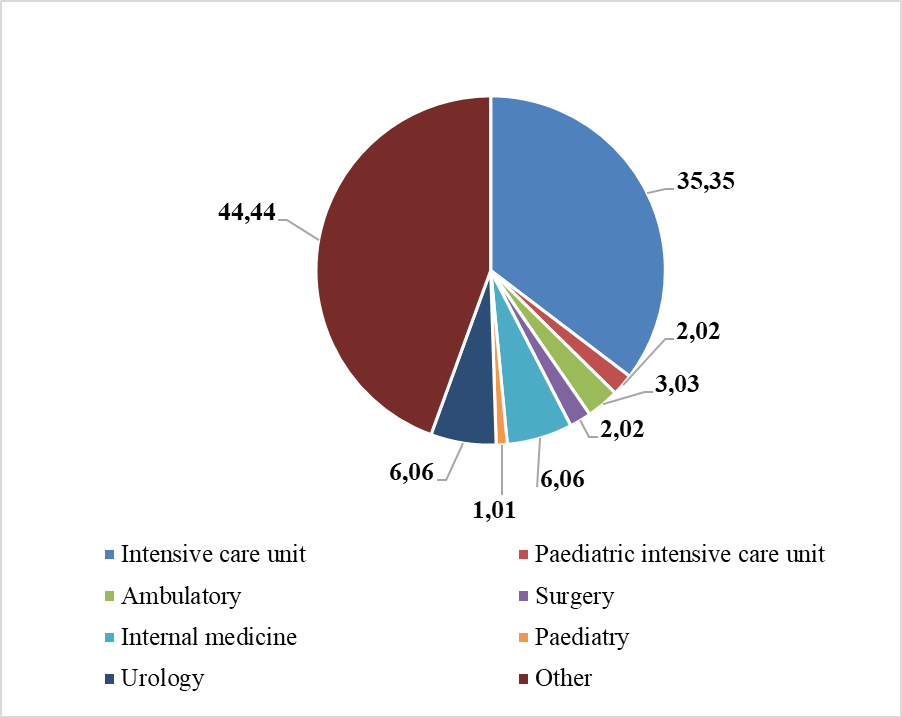


Figure S1. Distribution of the 99 *Klebsiella pneumoniae* isolates according to the hospital departments, in percentage.


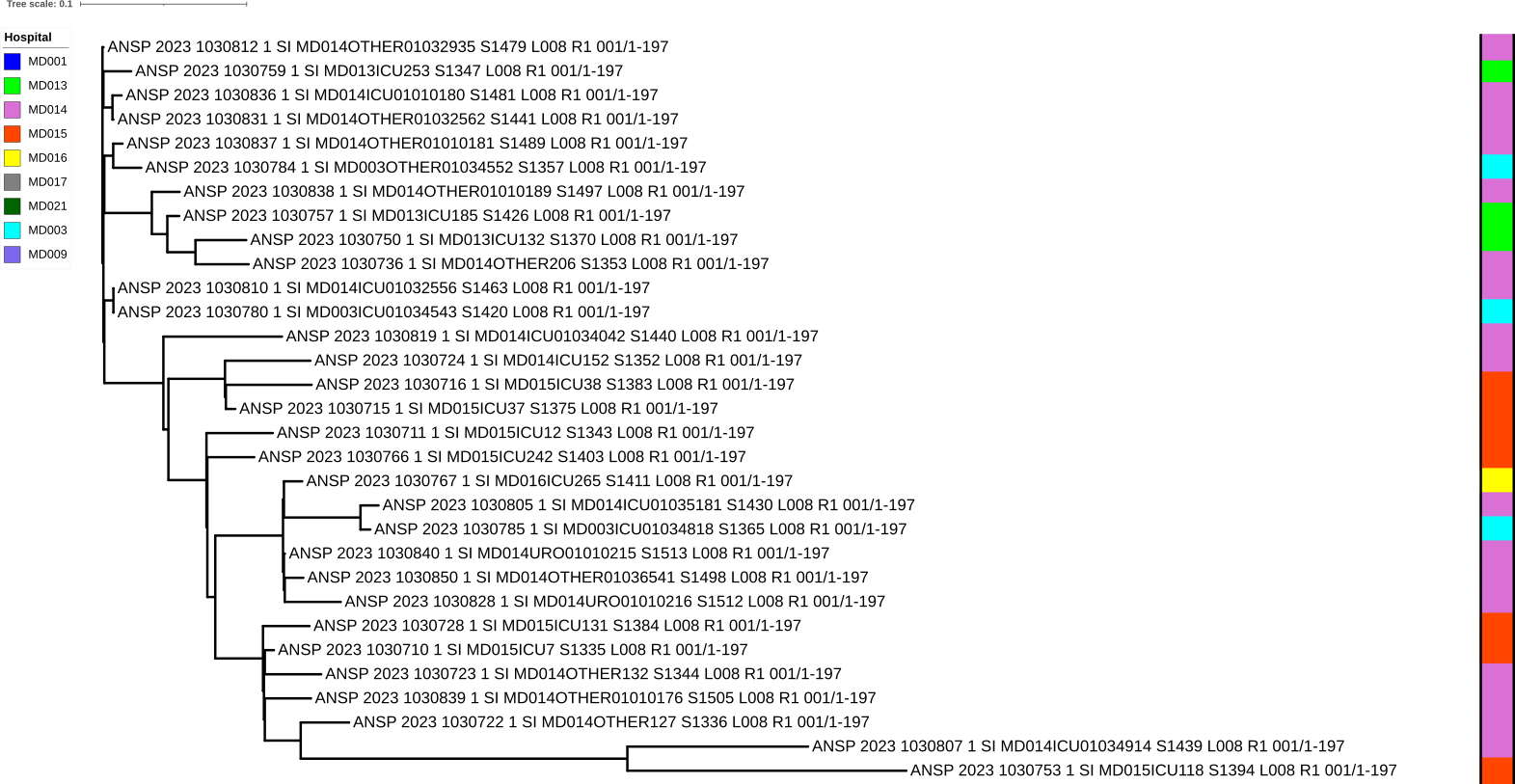

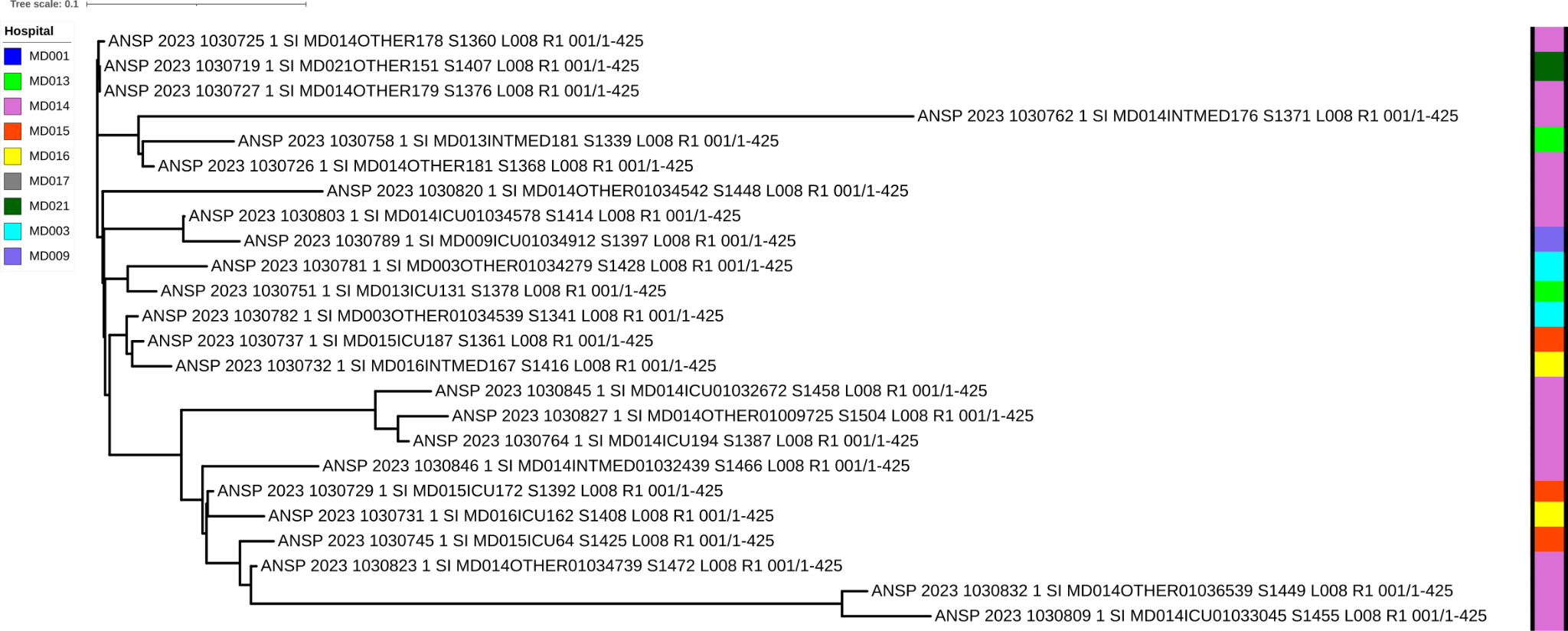

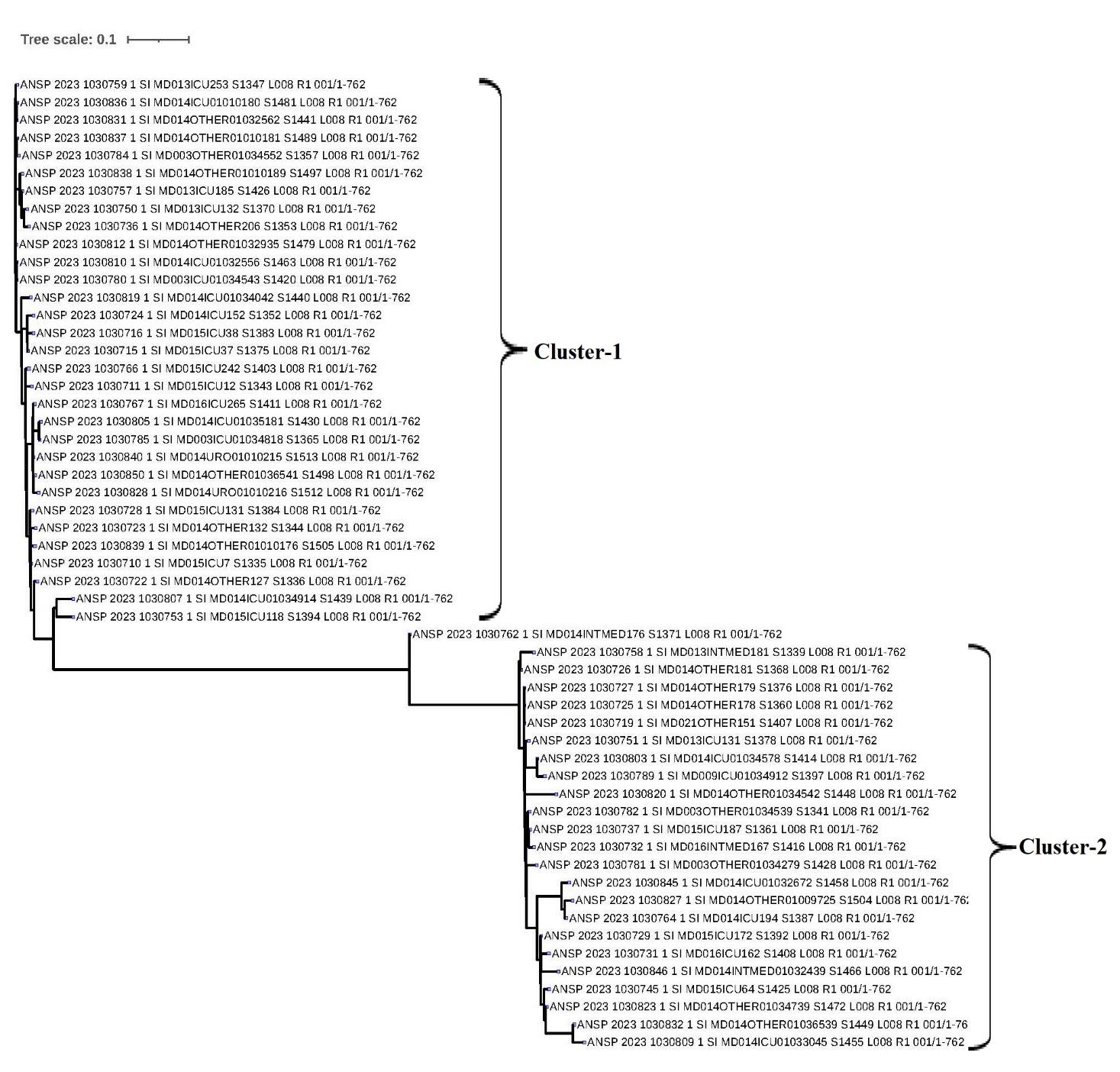
Figure S2. **Phylogenetic tree of the 56 *Klebsiella pneumoniae* ST395 strains. Top all 56 strains. Bottowm each cluster independently**

Figure 3. **Distribution of plasmids identified in *Klebsiella pneumoniae* using the online tool PlasmidFinder.**

SUPPLEMENTARY TABLES

Table S1**. Clinical information about isolates of *K. pneumoniae***

| **No.** | **Isolate ID** | **ENA accession number** | **Year of isolation** | **Biosubstrate** | **Resistance machanism** | **Isolate origin** | **Hospital department** |
| --- | --- | --- | --- | --- | --- | --- | --- |
|  | 1030710 | ERS24931633 | 2020 | Blood | ESBL, CPKP | Hospital | Intensive care unit |
|  | 1030711 | ERS24931634 | 2020 | Blood | CPKP | Hospital | Intensive care unit |
|  | 1030712 | ERS24931635 | 2023 | Blood | Negative** | Hospital | Intensive care unit |
|  | 1030713 | ERS24931636 | 2020 | CSF | CPKP | Hospital | Intensive care unit |
|  | 1030714 | ERS24931637 | 2020 | CSF | CPKP | Hospital | Intensive care unit |
|  | 1030715 | ERS24931638 | 2020 | Blood | CPKP | Hospital | Intensive care unit |
|  | 1030716 | ERS24931639 | 2020 | Blood | CPKP | Hospital | Intensive care unit |
|  | 1030717 | ERS24931640 | 2023 | Blood | CPKP | Hospital | Intensive care unit |
|  | 1030718 | ERS24931641 | 2020 | Blood | CPKP | Hospital | Intensive care unit |
|  | 1030719 | ERS24931642 | 2020 | Blood | CPKP | Hospital | Other* |
|  | 1030776 | ERS24931699 | 2020 | Urine | ESBL | Ambulatory | Consultative |
|  | 1030777 | ERS24931700 | 2020 | Urine | ESBL | Ambulatory | Consultative |
|  | 1030722 | ERS24931645 | 2021 | Blood | CPKP | Hospital | Other* |
|  | 1030723 | ERS24931646 | 2021 | Blood | CPKP | Hospital | Other* |
|  | 1030724 | ERS24931647 | 2023 | Blood | CPKP | Hospital | Other* |
|  | 1030725 | ERS24931648 | 2021 | Blood | CPKP | Hospital | Other* |
|  | 1030726 | ERS24931649 | 2021 | Blood | CPKP | Hospital | Other* |
|  | 1030727 | ERS24931650 | 2021 | Blood | CPKP | Hospital | Other* |
|  | 1030728 | ERS24931651 | 2021 | Blood | CPKP | Hospital | Other* |
|  | 1030729 | ERS24931652 | 2021 | Blood | CPKP | Hospital | Other* |
|  | 1030730 | ERS24931653 | 2023 | Blood | ESBL | Hospital | Other* |
|  | 1030731 | ERS24931654 | 2021 | Blood | CPKP | Hospital | Other* |
|  | 1030732 | ERS24931655 | 2021 | Blood | CPKP | Hospital | Other* |
|  | 1030733 | ERS24931656 | 2021 | Blood | ESBL | Hospital | Other* |
|  | 1030734 | ERS24931657 | 2023 | Blood | ESBL | Hospital | Other* |
|  | 1030736 | ERS24931659 | 2022 | Blood | CPKP | Hospital | Other* |
|  | 1030737 | ERS24931660 | 2021 | Blood | CPKP | Hospital | Other* |
|  | 1030741 | ERS24931664 | 2022 | Blood | CPKP | Hospital | Other* |
|  | 1030744 | ERS24931667 | 2023 | Blood | CPKP | Hospital | Other* |
|  | 1030745 | ERS24931668 | 2023 | Blood | CPKP | Hospital | Other* |
|  | 1030750 | ERS24931673 | 2022 | Blood | CPKP | Hospital | Other* |
|  | 1030751 | ERS24931674 | 2022 | Blood | CPKP | Hospital | Other* |
|  | 1030752 | ERS24931675 | 2022 | Blood | ESBL | Hospital | Other* |
|  | 1030753 | ERS24931676 | 2022 | Blood | CPKP | Hospital | Intensive care unit |
|  | 1030754 | ERS24931677 | 2022 | CSF | ESBL | Hospital | Paediatry |
|  | 1030755 | ERS24931678 | 2022 | Blood | CPKP | Hospital | Intensive care unit |
|  | 1030756 | ERS24931679 | 2022 | Blood | CPKP | Hospital | Intensive care unit |
|  | 1030757 | ERS24931680 | 2022 | Blood | CPKP | Hospital | Intensive care unit |
|  | 1030758 | ERS24931681 | 2022 | Blood | CPKP | Hospital | Internal medicine |
|  | 1030759 | ERS24931682 | 2022 | Blood | CPKP | Hospital | Intensive care unit |
|  | 1030760 | ERS24931683 | 2022 | Blood | CPKP | Hospital | Intensive care unit |
|  | 1030762 | ERS24931685 | 2022 | Blood | CPKP | Hospital | Internal medicine |
|  | 1030763 | ERS24931686 | 2022 | Blood | CPKP | Hospital | Intensive care unit |
|  | 1030764 | ERS24931687 | 2022 | Blood | CPKP | Hospital | Intensive care unit |
|  | 1030765 | ERS24931688 | 2022 | Blood | ESBL | Hospital | Intensive care unit |
|  | 1030766 | ERS24931689 | 2022 | Blood | CPKP | Hospital | Intensive care unit |
|  | 1030767 | ERS24931690 | 2022 | Blood | CPKP | Hospital | Intensive care unit |
|  | 1030768 | ERS24931691 | 2022 | Blood | ESBL | Hospital | Paediatric intensive care unit |
|  | 1030769 | ERS24931692 | 2022 | Blood | ESBL | Hospital | Paediatric intensive care unit |
|  | 1030780 | ERS24931703 | 2023 | Urine | CPKP | Hospital | Intensive care unit |
|  | 1030781 | ERS24931704 | 2023 | Urine | CPKP | Hospital | Other* |
|  | 1030782 | ERS24931705 | 2023 | Urine | CPKP | Hospital | Other* |
|  | 1030783 | ERS24931706 | 2023 | Urine | ESBL | Hospital | Other* |
|  | 1030784 | ERS24931707 | 2023 | Urine | ESBL | Hospital | Other* |
|  | 1030785 | ERS24931708 | 2023 | Urine | CPKP | Hospital | Intensive care unit |
|  | 1030789 | ERS24931712 | 2023 | Urine | CPKP | Hospital | Intensive care unit |
|  | 1030803 | ERS24931726 | 2023 | Urine | ESBL | Hospital | Intensive care unit |
|  | 1030804 | ERS24931727 | 2023 | Urine | ESBL | Hospital | Other* |
|  | 1030805 | ERS24931728 | 2023 | Urine | CPKP | Hospital | Intensive care unit |
|  | 1030806 | ERS24931729 | 2023 | Urine | ESBL, CPKP | Hospital | Internal medicine |
|  | 1030807 | ERS24931730 | 2023 | Urine | CPKP | Ambulatory | Consultative |
|  | 1030809 | ERS24931732 | 2023 | Urine | CPKP | Hospital | Intensive care unit |
|  | 1030810 | ERS24931733 | 2023 | Urine | CPKP | Hospital | Intensive care unit |
|  | 1030811 | ERS24931734 | 2023 | Urine | CPKP | Hospital | Intensive care unit |
|  | 1030812 | ERS24931735 | 2023 | Urine | CPKP | Hospital | Other* |
|  | 1030813 | ERS24931736 | 2023 | Urine | CPKP | Hospital | Urology |
|  | 1030814 | ERS24931737 | 2023 | Urine | CPKP | Hospital | Intensive care unit |
|  | 1030816 | ERS24931739 | 2023 | Urine | CPKP | Hospital | Intensive care unit |
|  | 1030819 | ERS24931742 | 2023 | Urine | CPKP | Hospital | Intensive care unit |
|  | 1030820 | ERS24931743 | 2023 | Urine | CPKP | Hospital | Other* |
|  | 1030821 | ERS24931744 | 2023 | Urine | CPKP | Hospital | Other* |
|  | 1030822 | ERS24931745 | 2023 | Urine | ESBL | Hospital | Intensive care unit |
|  | 1030823 | ERS24931746 | 2023 | Urine | CPKP | Hospital | Other* |
|  | 1030824 | ERS24931747 | 2023 | Urine | ESBL | Hospital | Other* |
|  | 1030825 | ERS24931748 | 2023 | Urine | ESBL | Hospital | Other* |
|  | 1030826 | ERS24931749 | 2023 | Urine | ESBL | Hospital | Urology |
|  | 1030827 | ERS24931750 | 2023 | Urine | CPKP | Hospital | Other* |
|  | 1030828 | ERS24931751 | 2023 | Urine | CPKP | Hospital | Urology |
|  | 1030829 | ERS24931752 | 2023 | Urine | CPKP | Hospital | Other* |
|  | 1030830 | ERS24931753 | 2023 | Urine | Negativ** | Hospital | Other* |
|  | 1030831 | ERS24931754 | 2023 | Urine | CPKP | Hospital | Other* |
|  | 1030832 | ERS24931755 | 2021 | Urine | CPKP | Hospital | Other* |
|  | 1030833 | ERS24931756 | 2023 | Urine | ESBL | Hospital | Surgical |
|  | 1030834 | ERS24931757 | 2023 | Urine | CPKP | Hospital | Other* |
|  | 1030835 | ERS24931758 | 2023 | Urine | Negativ** | Hospital | Surgical |
|  | 1030836 | ERS24931759 | 2023 | Urine | CPKP | Hospital | Intensive care unit |
|  | 1030837 | ERS24931760 | 2023 | Urine | CPKP | Hospital | Other* |
|  | 1030838 | ERS24931761 | 2023 | Urine | CPKP | Hospital | Other* |
|  | 1030839 | ERS24931762 | 2023 | Urine | ESBL | Hospital | Other* |
|  | 1030840 | ERS24931763 | 2023 | Urine | CPKP | Hospital | Urology |
|  | 1030841 | ERS24931764 | 2023 | Urine | CPKP | Hospital | Urology |
|  | 1030842 | ERS24931765 | 2023 | Urine | CPKP | Hospital | Other* |
|  | 1030844 | ERS24931767 | 2023 | Urine | ESBL | Hospital | Urology |
|  | 1030845 | ERS24931768 | 2023 | Urine | CPKP | Hospital | Intensive care unit |
|  | 1030846 | ERS24931769 | 2023 | Urine | CPKP | Hospital | Internal medicine |
|  | 1030850 | ERS24931773 | 2023 | Urine | CPKP | Hospital | Other* |
|  | 1030851 | ERS24931774 | 2023 | Urine | ESBL | Hospital | Internal medicine |
|  | 1030852 | ERS24931775 | 2023 | Urine | CPKP | Hospital | Internal medicine |
|  | 1030853 | ERS24931776 | 2023 | Urine | CPKP | Hospital | Internal medicine |

* Other departments: Urology, epileptology, admission ward, traumatology etc.

** Negative result – not confirmed resistance mechanism by ESBL/carbapenemase production

Table S2. **Colistin susceptibility by MLST type of tested strains**

| **Year of identification** | **Isolate ID** | **Susceptibility to colistin** | **ST** |
| --- | --- | --- | --- |
| 2020 | 1030711 | Susceptible | 395 |
| 2020 | 1030713 | Susceptible | 395 |
| 2020 | 1030715 | Susceptible | 11 |
| 2020 | 1030716 | Susceptible | 23 |
| 2023 | 1030717 | Susceptible | 11 |
| 2020 | 1030718 | Susceptible | 395 |
| 2020 | 1030719 | Susceptible | 395 |
| 2021 | 1030724 | Susceptible | 1026 |
| 2021 | 1030725 | Susceptible | 377 |
| 2021 | 1030726 | Resistant | 395 |
| 2021 | 1030727 | Resistant | 395 |
| 2023 | 1030730 | Susceptible | 23 |
| 2021 | 1030731 | Susceptible | 395 |
| 2021 | 1030732 | Susceptible | 395 |
| 2023 | 1030734 | Susceptible | 395 |
| 2023 | 1030736 | Susceptible | 395 |
| 2021 | 1030737 | Resistant | 395 |
| 2022 | 1030750 | Susceptible | 395 |
| 2022 | 1030751 | Susceptible | 395 |
| 2023 | 1030809 | Susceptible | 395 |
| 2023 | 1030810 | Susceptible | 14 |
| 2023 | 1030811 | Susceptible | 395 |
| 2023 | 1030812 | Susceptible | 395 |
| 2023 | 1030813 | Susceptible | 37 |
| 2023 | 1030814 | Susceptible | 340 |
| 2023 | 1030816 | Susceptible | 395 |
| 2023 | 1030825 | Resistant | 395 |
| 2023 | 1030826 | Susceptible | 101 |
| 2023 | 1030827 | Susceptible | 23 |
| 2023 | 1030828 | Susceptible | 395 |
| 2023 | 1030829 | Susceptible | 395 |
| 2023 | 1030831 | Susceptible | 395 |
| 2023 | 1030836 | Resistant | 11 |
| 2023 | 1030837 | Susceptible | 395 |
| 2023 | 1030838 | Susceptible | 1026 |
| 2023 | 1030839 | Susceptible | 377 |
| 2023 | 1030840 | Susceptible | 377 |
| 2023 | 1030841 | Susceptible | 395 |
| 2023 | 1030842 | Susceptible | 395 |
| 2023 | 1030845 | Susceptible | 395 |
| 2023 | 1030846 | Susceptible | 377 |
| 2023 | 1030853 | Susceptible | 395 |

Table S3. **Structure of *K. pneumoniae* isolates tested by PCR in concordance with MLST and identified resistance genes**

| **Isolate ID** | **Biosubstrate** | **Resistance genes, ResFinder** | **ST** |
| --- | --- | --- | --- |
| 1030710 | Blood | ant(2'')-Ia, ant(3'')-Ia, aadA1, aac(6')-Ib-cr, *bla*OXA-1, *bla*OXA-48, *bla*SHV-158, *bla*TEM-1B, *bla*CTX-M-15, *bla*SHV-182, fosA, catA1, qnrS1, OqxA, OqxB, sul1, tet(A), dfrA1, qacE | 395 |
| 1030711 | Blood | ant(2'')-Ia, ant(3'')-Ia, rmtC, aac(6')-Ib-cr, *bla*OXA-1, *bla*OXA-48, *bla*TEM-1B, *bla*NDM-1, *bla*CTX-M-15, *bla*SHV-11, *bla*SHV-182, fosA, catA1, qnrS, OqxA, OqxB, sul1, tet(A), dfrA1, qacE | 395 |
| 1030712 | Blood | rmtF, aac(6')-Ib-Hangzhou, aadA2, aph(3')-Ia, *bla*SHV-182, *bla*SHV-159, *bla*SHV-158, *bla*CTX-M-15, fosA6, mph(A), OqxB, OqxA, qnrB1, ARR-2, sul1, dfrA12 | 11 |
| 1030713 | CSF | aac(6')-Ib-cr, *bla*OXA-1, *bla*SHV-33, *bla*SHV-45, *bla*SHV-51, *bla*CTX-M-55, *bla*LAP-2, fosA, catA2, qnrS1, aac(6')-Ib-cr, aac(6')-Ib-cr, OqxA, OqxB, tet(A), qacE | 23 |
| 1030714 | CSF | aadA2, aph(3')-Ia, rmtF, aac(6')-Ib-cr, aac(3)-IIa, *bla*OXA-1, *bla*OXA-48, *bla*SHV-158, *bla*CTX-M-15, *bla*SHV-182, *bla*LAP-2, fosA5, fosA, mph(A), catA2, qnrS1, OqxA, OqxB, ARR-2, ARR-3, sul1, dfrA12, qacE | 11 |
| 1030715 | Blood | rmtC, aac(6')-Ib-cr, *bla*OXA-1, *bla*SHV-158, *bla*TEM-1B, *bla*NDM-1, *bla*CTX-M-15, *bla*SHV-182, fosA, qnrS1, OqxA, OqxB, sul1, tet(A), dfrA1, qacE | 395 |
| 1030716 | Blood | ant(2'')-Ia, ant(3'')-Ia, rmtC, aac(6')-Ib-cr, *bla*OXA-1, *bla*OXA-48 ,*bla*TEM-1B, *bla*NDM-1, *bla*CTX-M-15, *bla*SHV-182, fosA, catA1, qnrS1, OqxA, OqxB, sul1, tet(A), dfrA1, qacE | 395 |
| 1030717 | Blood | aac(6')-Ib-cr, aac(3)-IIa, *bla*OXA-1, *bla*SHV-33, *bla*CTX-M-15, *bla*LAP-2, fosA, catA2, qnrS1, aac(6')-Ib-cr, OqxA, OqxB | 1026 |
| 1030718 | Blood | aph(3')-VI, aph(3')-Via, armA, aac(6')-Ib-cr, aac(3)-IIa, *bla*OXA-1, *bla*OXA-48, *bla*SHV-110, *bla*TEM-1B, *bla*CTX-M-15, fosA, msr(E), mph(E), catA1, OqxA, OqxB | 377 |
| 1030719 | Blood | aph(3')-VI, aph(3')-Via, rmtC, aac(6')-Ib-cr, *bla*OXA-1, *bla*OXA-48, *bla*SHV-158, *bla*TEM-1B, *bla*NDM-1, *bla*CTX-M-15, *bla*SHV-182, fosA, catA1, qnrS1, OqxA, OqxB, sul1, tet(A), dfrA1, qacE | 395 |
| 1030776 | Urine | *bla*SHV-85, fosA, fosA6, OqxA, OqxB | 395 |
| 1030777 | Urine | aac(6')-Ib-cr, aac(3)-IIa, *bla*OXA-1, *bla*OXA-72, *bla*SHV-45, *bla*SHV-51, *bla*CTX-M-55, *bla*LAP-2, fosA, catA2, qnrS1, OqxA, OqxB, tet(A) | **23** |
| 1030722 | Blood | ant(2'')-Ia, ant(3'')-Ia, aac(6')-Ib-cr, *bla*OXA-1, *bla*OXA-48, *bla*SHV-158, *bla*TEM-1B, *bla*CTX-M-15, *bla*SHV-182, fosA, fosA6, catA1, qnrS1, OqxA, OqxB, sul1, tet(A), dfrA1, qacE | 395 |
| 1030723 | Blood | ant(2'')-Ia, ant(3'')-Ia, aph(3')-Via, aac(6')-Ib-cr, *bla*OXA-1, *bla*OXA-48, *bla*TEM-1B, *bla*CTX-M-15, *bla*SHV-11, *bla*SHV-182, fosA, catA1, qnrS1, OqxA,OqxB, sul1, tet(A), dfrA1, qacE | 395 |
| 1030724 | Blood | rmtC, aac(6')-Ib-cr, *bla*OXA-1, *bla*TEM-1B, *bla*NDM-1, *bla*CTX-M-15, *bla*SHV-11, *bla*SHV-182, fosA, qnrS1, OqxA, OqxB, sul1, tet(A), dfrA1, qacE | 395 |
| 1030725 | Blood | aph(3')-VI, aph(3')-Via, rmtC, aac(6')-Ib-cr, *bla*OXA-1, *bla*OXA-48, *bla*TEM-1B, *bla*NDM-1, *bla*CTX-M-15, *bla*SHV-11, *bla*SHV-182, fosA, catA1, qnrS1, OqxA, OqxB, sul1, tet(A), dfrA1, qacE | 395 |
| 1030726 | Blood | aph(3')-VI, aph(3')-Via, rmtC, aac(6')-Ib-cr, *bla*OXA-1, *bla*OXA-48, *bla*TEM-1B, *bla*NDM-1, *bla*CTX-M-15, *bla*SHV-182, fosA, catA1, qnrS1, OqxA, OqxB, sul1, tet(A), dfrA1, qacE | 395 |
| 1030727 | Blood | aph(3')-VI, aac(6')-Ib, aph(3')-Via, rmtC, aac(6')-Ib-cr, *bla*OXA-1, *bla*OXA-48, *bla*SHV-158, *bla*TEM-1B, *bla*NDM-1, *bla*CTX-M-15, *bla*SHV-182, fosA, catA1, qnrS1, OqxA, OqxB, sul1, tet(A), dfrA1, qacE | 395 |
| 1030728 | Blood | aac(6')-Ib-cr, *bla*OXA-1, *bla*OXA-48, *bla*SHV-158, *bla*TEM-1B, *bla*CTX-M-15, *bla*SHV-182, fosA, qnrS1, OqxA, OqxB, sul1, tet(A), dfrA1, qacE | 395 |
| 1030729 | Blood | aac(6')-Ib-cr, aac(3)-IIa, *bla*OXA-1, *bla*OXA-48, *bla*TEM-1B, *bla*CTX-M-15, *bla*SHV-182, fosA, catA1, qnrS1, OqxA, OqxB, sul1, tet(A), dfrA1, qacE | 395 |
| 1030730 | Blood | aac(6')-Ib-cr, aac(3)-IIa, *bla*OXA-1, *bla*SHV-100, *bla*TEM-1B, *bla*CTX-M-15, *bla*LAP-2, fosA, fosA6, catA2, qnrS1, OqxA, OqxB, dfrA14, qacE | 14 |
| 1030731 | Blood | aph(6)-Id, aac(6')-Ib, aac(6')-Ib-cr, aac(3)-IIa, *bla*OXA-1, *bla*OXA-48, *bla*TEM-1B, *bla*CTX-M-15, *bla*SHV-11, *bla*SHV-182, fosA, catA1, qnrS1, OqxA, OqxB, sul1, tet(A), dfrA1, qacE | 395 |
| 1030732 | Blood | rmtC, aac(6')-Ib-cr, aac(3)-IIa, *bla*OXA-1, *bla*OXA-48, *bla*TEM-1B, *bla*NDM-1, *bla*CTX-M-15, *bla*SHV-182, fosA, catA1, qnrS1, OqxA, OqxB, sul1, tet(A), dfrA1, qacE | 395 |
| 1030733 | Blood | aph(6)-Id, aac(6')-Ib-cr, aph(3'')-Ib, aac(3)-IIa, *bla*OXA-1, *bla*SHV-85, *bla*TEM-1B, *bla*CTX-M-15, fosA, fosA6, qnrB1, OqxA, OqxB, sul2, tet(A), dfrA14 | 37 |
| 1030734 | Blood | aadA2, aac(6')-Ib-cr, aac(3)-IIa, *bla*OXA-1, *bla*TEM-1B, *bla*CTX-M-15, *bla*SHV-182, fosA5, fosA, catA1, qnrS1, aac(6')-Ib-cr, OqxA, OqxB, sul1, tet(A), dfrA1, qacE | 340 |
| 1030736 | Blood | rmtC, aac(6')-Ib-cr*bla*OXA-1, *bla*OXA-48, *bla*TEM-1B, *bla*NDM-1, *bla*CTX-M-15, *bla*SHV-182, fosA, catA1, qnrS1, OqxA, OqxB, sul1, tet(A), dfrA1 | 395 |
| 1030737 | Blood | rmtC, aac(6')-Ib-cr, aac(3)-IIa, *bla*OXA-1, *bla*OXA-48, *bla*TEM-1B, *bla*NDM-1, *bla*CTX-M-15, *bla*SHV-11, *bla*SHV-182, fosA, catA1, qnrS1, OqxA, OqxB, sul1, tet(A), dfrA1 | 395 |
| 1030741 | Blood | aadA5, *bla*CTX-M-15, mph(A), mdf(A), sul1, tet(A), dfrA17, qacE, sitABCD | 101 |
| 1030744 | Blood | ant(2'')-Ia, ant(3'')-Ia, aac(6')-Ib-cr, aac(3)-IIa, *bla*OXA-1, *bla*OXA-48, *bla*SHV-51, *bla*TEM-1B, *bla*CTX-M-55, *bla*LAP-2, fosA, catA1, catA2, qnrS1, OqxA, OqxB | **23** |
| 1030745 | Blood | aac(6')-Ib-cr, aac(3)-IIa, *bla*OXA-1, *bla*OXA-48, *bla*TEM-1B, *bla*CTX-M-15, *bla*SHV-11, *bla*SHV-182, fosA, catA1, qnrS1, OqxA, OqxB, sul1, tet(A), dfrA1, qacE | 395 |
| 1030750 | Blood | ant(2'')-Ia, ant(3'')-Ia, rmtC, aac(6')-Ib-cr, aac(3)-IIa, *bla*OXA-1, *bla*OXA-48, *bla*SHV-159, *bla*TEM-1B, *bla*NDM-1, *bla*CTX-M-15, *bla*SHV-182, fosA, catA1, qnrS1, OqxA, OqxB, sul1, tet(A), dfrA1 | 395 |
| 1030751 | Blood | rmtC, aac(6')-Ib-cr, aac(3)-IIa, *bla*OXA-1, *bla*OXA-48, *bla*SHV-158, *bla*TEM-1B, *bla*NDM-1, *bla*CTX-M-15, *bla*SHV-182, fosA, catA1, qnrS1, OqxA, OqxB, sul1, tet(A), dfrA1, qacE | 395 |
| 1030752 | Blood | aadA2, aph(3')-Ia, rmtF, aac(6')-Ib-cr, aac(3)-IIa, *bla*SHV-158, *bla*CTX-M-15, *bla*SHV-182, fosA5, fosA, catA2, qnrS1, OqxA, OqxB, dfrA12 | 11 |
| 1030753 | Blood | aac(6')-Ib-cr, *bla*OXA-1, *bla*OXA-48, *bla*TEM-1B, *bla*CTX-M-15, *bla*SHV-182, fosA, qnrS1, OqxA, OqxB, sul1, tet(A), dfrA1, qacE | 395 |
| 1030754 | CSF | aac(6')-Ib, aac(6')-Ib-cr, aac(3)-IIa, *bla*OXA-1, *bla*SHV-33, *bla*CTX-M-15, *bla*LAP-2, fosA, catA2, qnrS1, OqxA, OqxB | 1026 |
| 1030755 | Blood | aph(3')-VI, aac(6')-Ib, aph(3')-Via, armA, aac(6')-Ib-cr, aac(3)-IIa, *bla*OXA-1, *bla*OXA-48, *bla*SHV-110, *bla*TEM-1B, *bla*TEM-183, *bla*CTX-M-15, fosA, msr(E), mph(E), catA1, OqxA, OqxB | 377 |
| 1030756 | Blood | aac(6')-Ib, aph(3')-VI, aph(3')-Via, armA, aac(6')-Ib-cr, aac(3)-IIa, *bla*OXA-1, *bla*OXA-48, *bla*SHV-110, *bla*TEM-1B, *bla*CTX-M-15, fosA, msr(E), mph(E), catA1, OqxA, OqxB | 377 |
| 1030757 | Blood | ant(2'')-Ia, ant(3'')-Ia, aadA1, rmtC, aac(6')-Ib-cr, *bla*OXA-1, *bla*OXA-48, *bla*TEM-1B, *bla*NDM-1, *bla*CTX-M-15, *bla*SHV-182, fosA, catA1, qnrS1, OqxA, OqxB, sul1, tet(A), dfrA1, qacE | 395 |
| 1030758 | Blood | aac(6')-Ib-cr, *bla*OXA-1, *bla*OXA-48, *bla*SHV-158, *bla*TEM-1B, *bla*CTX-M-15, *bla*SHV-182, fosA, catA1, qnrS1, OqxA, OqxB, sul1, tet(A), dfrA1, qacE | 395 |
| 1030759 | Blood | ant(2'')-Ia, ant(3'')-Ia, rmtC, aac(6')-Ib-cr, *bla*OXA-1, *bla*OXA-48, *bla*TEM-1B, *bla*NDM-1, *bla*CTX-M-15, *bla*SHV-11, *bla*SHV-182, fosA, catA1, qnrS1, OqxA, OqxB,, sul1, tet(A), dfrA1, qacE | 395 |
| 1030760 | Blood | aph(3')-VI, aac(6')-Ib, aph(3')-Via, armA, aac(6')-Ib-cr, aac(3)-IIa, *bla*OXA-1, *bla*OXA-48, *bla*SHV-110, *bla*TEM-1B, *bla*CTX-M-15, fosA, msr(E), mph(E), catA1, OqxA, OqxB | 377 |
| 1030762 | Blood | aph(3')-VI, aph(3')-Via, rmtC, aac(6')-Ib-cr, aac(3)-IIa, *bla*OXA-1, *bla*OXA-48, *bla*SHV-158, *bla*TEM-1B, *bla*NDM-1, *bla*CTX-M-15, *bla*SHV-182, *bla*LAP-2, fosA, fosA7, catA1, catA2, qnrS1, OqxA, OqxB, sul1, tet(A), dfrA1, qacE | 395 |
| 1030763 | Blood | aac(6')-Ib-cr, aac(3)-IIa, *bla*OXA-1, *bla*SHV-81, *bla*SHV-110, *bla*CTX-M-15, *bla*SHV-11, *bla*LAP-2, fosA, fosA7, fosA6, catA2, qnrS1, OqxA, OqxB | 6381 |
| 1030764 | Blood | aac(6')-Ib-cr, aac(3)-IIa, *bla*OXA-1, *bla*OXA-48, *bla*SHV-158, *bla*CTX-M-15, *bla*SHV-182, fosA, catA1, OqxA, OqxB, sul1, tet(A), dfrA1, qacE | 395 |
| 1030765 | Blood | aph(3')-VI, aac(6')-Ib, aph(3')-Via, armA, aac(6')-Ib-cr, aac(3)-IIa, *bla*OXA-1, *bla*OXA-48, *bla*SHV-110, *bla*TEM-1B, *bla*CTX-M-15, fosA5, fosA, msr(E), mph(E), catA1, OqxA, OqxB | 377 |
| 1030766 | Blood | ant(2'')-Ia, ant(3'')-Ia, rmtC, aac(6')-Ib-cr, *bla*OXA-1, *bla*OXA-48, *bla*SHV-158, *bla*TEM-1B, *bla*NDM-1, *bla*CTX-M-15, *bla*SHV-182, fosA, catA1, qnrS1, aac(6')-Ib-cr, OqxA, OqxB, sul1, tet(A), dfrA1, qacE, OqxA, OqxB | 25 |
| 1030767 | Blood | ant(2'')-Ia, ant(3'')-Ia, rmtC, aac(6')-Ib-cr, *bla*OXA-1, *bla*OXA-48, *bla*SHV-158, *bla*TEM-1B, *bla*NDM-1, *bla*CTX-M-15, *bla*SHV-182, fosA, catA1, qnrS1, OqxA, OqxB, sul1, tet(A), dfrA1, qacE | 395 |
| 1030768 | Blood | aac(6')-Ib-cr, aac(3)-IIa, *bla*OXA-1, *bla*CTX-M-15, *bla*SHV-196, *bla*LAP-2, fosA, catA2, qnrS1, OqxA, OqxB | 1026 |
| 1030769 | Blood | aac(6')-Ib-cr, aac(3)-IIa, *bla*OXA-1, *bla*CTX-M-15, *bla*SHV-196, *bla*LAP-2, fosA, catA2, qnrS1, OqxA, OqxB | 1026 |
| 1030780 | Urine | ant(2'')-Ia, ant(3'')-Ia, rmtC, *bla*OXA-48, *bla*OXA-72, *bla*SHV-158, *bla*TEM-1B, *bla*NDM-1, *bla*SHV-182, fosA, catA1, qnrS1, OqxA, OqxB, sul1, tet(A), dfrA1, qacE | 395 |
| 1030781 | Urine | rmtC, aac(6')-Ib-cr, *bla*OXA-1, *bla*OXA-48, *bla*SHV-158, *bla*TEM-1B, *bla*NDM-1, *bla*CTX-M-15, *bla*SHV-182, fosA, catA1, qnrS1, OqxA, OqxB, sul1, tet(A), dfrA1, qacE | 395 |
| 1030782 | Urine | rmtC, aac(6')-Ib-cr, *bla*OXA-1, *bla*OXA-48, *bla*TEM-1B, *bla*NDM-1, *bla*CTX-M-15, *bla*SHV-182, fosA, catA1, qnrS1, sul1, tet(A), dfrA1 | 395 |
| 1030783 | Urine | ant(2'')-Ia, ant(3'')-Ia, aac(6')-Ib-cr, aac(3)-IIa, *bla*OXA-1, *bla*SHV-33, *bla*SHV-45, *bla*SHV-51, *bla*CTX-M-55, *bla*LAP-2, fosA, catA1, catA2, qnrS1, OqxA, OqxB, sul1, tet(A), qacE | **23** |
| 1030784 | Urine | ant(2'')-Ia, ant(3'')-Ia, rmtC, aac(6')-Ib-cr, *bla*OXA-1, *bla*OXA-48, *bla*OXA-72, *bla*TEM-1B, *bla*NDM-1, *bla*CTX-M-15, *bla*SHV-11, *bla*SHV-182, fosA, catA1, qnrS1, OqxA, OqxB, sul1, tet(A), dfrA1, qacE | 395 |
| 1030785 | Urine | ant(2'')-Ia, ant(3'')-Ia, rmtC, aac(6')-Ib-cr, *bla*OXA-1, *bla*OXA-48, *bla*OXA-72, *bla*TEM-1B, *bla*NDM-1, *bla*CTX-M-15, *bla*SHV-11, *bla*SHV-182, fosA, fosA6, catA1, qnrS1, OqxA, OqxB, sul1, tet(A), dfrA1, qacE | 395 |
| 1030789 | Urine | rmtC, aac(6')-Ib-cr, aac(3)-IIa, *bla*OXA-1, *bla*OXA-48, *bla*TEM-1B, *bla*NDM-1, *bla*SHV-182, fosA, catA1, qnrS1, sul1, tet(A), dfrA1 | 395 |
| 1030803 | Urine | rmtC, aac(6')-Ib-cr, aac(3)-IIa, *bla*OXA-1, *bla*OXA-48, *bla*SHV-158, *bla*TEM-1B, *bla*NDM-1, *bla*CTX-M-15, *bla*SHV-182, fosA, catA1, qnrS1, OqxA, OqxB, sul1, tet(A), dfrA1, qacE | 395 |
| 1030804 | Urine | aph(3')-Ia, aac(6')-Ib-cr, *bla*OXA-1, *bla*SHV-158, *bla*TEM-1B, *bla*CTX-M-15, *bla*SHV-182, fosA5, fosA, mph(A), catA1, qnrS1, OqxA, OqxB, sul1, tet(A), dfrA1, qacE | 340 |
| 1030805 | Urine | ant(2'')-Ia, ant(3'')-Ia, rmtC, aac(6')-Ib-cr, *bla*OXA-1, *bla*OXA-48, *bla*SHV-158, *bla*TEM-1B, *bla*NDM-1, *bla*CTX-M-15, *bla*SHV-182, fosA, catA1, qnrS1, OqxA, OqxB, sul1, tet(A), dfrA1, qacE | 395 |
| 1030806 | Urine | aac(6')-Ib, aadA1, armA, *bla*OXA-9, *bla*OXA-48, *bla*TEM-1B, *bla*CTX-M-15, *bla*SHV-11, *bla*SHV-12, fosA, msr(E), ere(B), mph(E), catA1, OqxA, OqxB | 147 |
| 1030807 | Urine | ant(2'')-Ia, ant(3'')-Ia, aac(6')-Ib-cr, *bla*OXA-1, *bla*OXA-48, *bla*TEM-1B, *bla*CTX-M-15, *bla*SHV-182, fosA, catA1, qnrS1, OqxA, OqxB, sul1, tet(A), dfrA1, qacE | 395 |
| 1030809 | Urine | aph(3')-VIa, aac(6')-Ib-cr, aac(3)-IIa, *bla*OXA-1, *bla*OXA-48, *bla*TEM-1B, *bla*CTX-M-15, *bla*SHV-11, *bla*SHV-182, fosA, catA1, qnrS1, OqxA, OqxB, sul1, tet(A), dfrA1, qacE | 395 |
| 1030810 | Urine | ant(2'')-Ia, ant(3'')-Ia, rmtC, aac(6')-Ib-cr, *bla*OXA-1, *bla*OXA-48, *bla*SHV-158, *bla*TEM-1B, *bla*NDM-1, *bla*CTX-M-15, *bla*SHV-182, fosA, catA1, qnrS1, OqxA, OqxB, sul1, tet(A), dfrA1, qacE | 395 |
| 1030811 | Urine | aph(3')-VI, aph(3')-Ia, aph(3')-VIa, armA, aac(6')-Ib-cr, *bla*OXA-1, *bla*OXA-48, *bla*SHV-158, *bla*TEM-1B, *bla*CTX-M-15, *bla*SHV-182, fosA5, fosA, msr(E), mph(A), mph(E), catA1, qnrS1, OqxA, OqxB, sul1, tet(A), dfrA1, qacE | 340 |
| 1030812 | Urine | ant(2'')-Ia, aac(6')-Ib, ant(3'')-Ia, rmtC, aac(6')-Ib-cr, *bla*OXA-1, *bla*OXA-48, *bla*SHV-158, *bla*TEM-1B, *bla*NDM-1, *bla*CTX-M-15, *bla*SHV-182, fosA, catA1, qnrS1, OqxA, OqxB, sul1, tet(A), dfrA1, qacE | 395 |
| 1030813 | Urine | aac(6')-Ib, armA, aac(6')-Ib-cr, aac(3)-IIa, *bla*OXA-1, *bla*OXA-48, *bla*SHV-110, *bla*TEM-1B, *bla*CTX-M-15, fosA, msr(E), mph(E), catA1, OqxA, OqxB | 377 |
| 1030814 | Urine | aph(3')-VI, aac(6')-Ib, aph(3')-VIa, armA, aac(6')-Ib-cr, aac(3)-IIa, *bla*OXA-1, *bla*OXA-48, *bla*SHV-110, *bla*TEM-1B, *bla*CTX-M-15, fosA5, fosA, msr(E), mph(E), catA1, OqxA OqxB | 377 |
| 1030816 | Urine | aph(3')-VI, aph(3')-VIa, armA, rmtC, aac(6')-Ib-cr, *bla*OXA-1, *bla*OXA-48, *bla*SHV-110, *bla*TEM-1B, *bla*NDM-1, *bla*CTX-M-15, fosA5, fosA, msr(E), mph(E), catA1, qnrS1, OqxA, OqxB, sul1, tet(A), dfrA1, qacE | 377 |
| 1030819 | Urine | rmtC, aac(6')-Ib-cr, *bla*OXA-1, *bla*OXA-48, *bla*TEM-1B, *bla*NDM-1, *bla*CTX-M-15, *bla*SHV-182, fosA, qnrS1, OqxA, OqxB, sul1, tet(A), dfrA1, qacE | 395 |
| 1030820 | Urine | rmtC, aac(6')-Ib-cr, aac(3)-IIa, *bla*OXA-1, *bla*OXA-48, *bla*TEM-1B, *bla*CTX-M-15, *bla*SHV-11, *bla*SHV-182, fosA, catA1, qnrS1, OqxA, OqxB, sul1, tet(A), dfrA1, qacE | 395 |
| 1030821 | Urine | aph(3')-Ia, armA, *bla*OXA-48, *bla*SHV-100, *bla*TEM-1B, *bla*CTX-M-15, fosA, fosA6, msr(E), mph(E), catA1, qnrS1, OqxA, OqxB, tet(A), dfrA30, qacE | 15 |
| 1030822 | Urine | aadA2, aph(3')-Ia, rmtF, *bla*CTX-M-15, *bla*SHV-182, fosA5, fosA, mph(A), qnrB1, OqxA, OqxB, ARR-2, ARR-3, sul1, dfrA1, dfrA12, qacE, OqxA, OqxB | 11 |
| 1030823 | Urine | aac(6')-Ib-cr, aac(3)-IIa, *bla*OXA-1, *bla*OXA-48, *bla*TEM-1B, *bla*CTX-M-15, *bla*SHV-11, *bla*SHV-182, fosA, catA1, qnrS1, OqxA, OqxB, sul1, tet(A), dfrA1, qacE | 395 |
| 1030824 | Urine | aph(6)-Id, aac(6')-Ib-cr, aph(3'')-Ib, aac(3)-IIa, *bla*OXA-1, *bla*SHV-100, *bla*CTX-M-15, fosA, fosA6, qnrB1, qnrS1, OqxA, OqxB, sul2, tet(A), dfrA14 | 15 |
| 1030825 | Urine | *bla*CTX-M-15, *bla*SHV-11, fosA, OqxA, OqxB | 147 |
| 1030826 | Urine | aph(6)-Id, aadA1, *bla*SHV-2a, *bla*SHV-5, *bla*SHV-40, *bla*SHV-89, *bla*SHV-12, fosA, catA1, qnrS1, OqxA, OqxB, sul1, tet(D), dfrA1, qacE | 1037 |
| 1030827 | Urine | aph(3')-VI, aph(3')-VIa, aac(6')-Ib-cr, aac(3)-IIa, *bla*OXA-1, *bla*OXA-48, *bla*SHV-158, *bla*CTX-M-15, *bla*SHV-182, fosA, catA1, OqxA, OqxB, sul1, tet(A), dfrA1, qacE | 395 |
| 1030828 | Urine | ant(2'')-Ia, ant(3'')-Ia, rmtC, aac(6')-Ib-cr, *bla*OXA-1, *bla*OXA-48, *bla*TEM-1B, *bla*NDM-1, *bla*CTX-M-15, *bla*SHV-182, fosA, catA1, qnrS1, OqxA, OqxB, sul1, tet(A), dfrA1 | 395 |
| 1030829 | Urine | aac(6')-Ib, armA, aac(6')-Ib-cr, aac(3)-IIa, *bla*OXA-1, *bla*OXA-48, *bla*OXA-72, *bla*SHV-110, *bla*TEM-1B, *bla*CTX-M-15, fosA5, fosA, msr(E), mph(E), catA1, OqxA, OqxB | 377 |
| 1030830 | Urine | aac(6')-Ib-cr, *bla*SHV-33, *bla*SHV-94, *bla*NDM-1, fosA, fosA6, OqxA, OqxB | 380 |
| 1030831 | Urine | ant(2'')-Ia, ant(3'')-Ia, rmtC, aac(6')-Ib-cr, *bla*OXA-1, *bla*OXA-48, *bla*SHV-159, *bla*TEM-1B, *bla*NDM-1, *bla*CTX-M-15, *bla*SHV-182, fosA, catA1, qnrS1, OqxA, OqxB, sul1, tet(A), dfrA1, qacE | 395 |
| 1030832 | Urine | aac(6')-Ib-cr, aac(3)-IIa, *bla*OXA-1, *bla*OXA-48, *bla*SHV-158, *bla*TEM-1B, *bla*CTX-M-15, *bla*SHV-182, fosA, catA1, qnrS1, OqxA, OqxB, sul1, tet(A), dfrA1, qacE | 395 |
| 1030833 | Urine | *bla*TEM-1B, *bla*CTX-M-15, fosA5, fosA, qepA1, aac(6')-Ib-cr, OqxA, OqxB | 101 |
| 1030834 | Urine | aac(6')-Ib, armA, aac(6')-Ib-cr, aac(3)-IIa, *bla*OXA-1, *bla*OXA-48, *bla*SHV-110, *bla*TEM-1B, *bla*CTX-M-15, fosA5, fosA, msr(E), mph(E), catA1, OqxA, OqxB | 377 |
| 1030835 | Urine | aac(6')-Ib, *bla*SHV-190, fosA, fosA6, fosA, aac(6')-Ib-cr, OqxA, OqxB | **23** |
| 1030836 | Urine | ant(2'')-Ia, ant(3'')-Ia, rmtC, aac(6')-Ib-cr, *bla*OXA-1, *bla*OXA-48, *bla*SHV-158, *bla*TEM-1B, *bla*NDM-1, *bla*CTX-M-15, *bla*SHV-182, fosA, catA1, qnrS1, OqxA, OqxB, sul1, tet(A), dfrA1, qacE | 395 |
| 1030837 | Urine | ant(2'')-Ia, ant(3'')-Ia, rmtC, aac(6')-Ib-cr, *bla*OXA-1, *bla*OXA-48, *bla*SHV-159, *bla*TEM-1B, *bla*NDM-1, *bla*CTX-M-15, *bla*SHV-182, fosA, catA1, qnrS1, OqxA, OqxB, sul1, tet(A), dfrA1, qacE | 395 |
| 1030838 | Urine | ant(2'')-Ia, ant(3'')-Ia, rmtC, aac(6')-Ib-cr, *bla*OXA-1, *bla*OXA-48, *bla*TEM-1B, *bla*NDM-1, *bla*CTX-M-15, *bla*SHV-11, *bla*SHV-182, fosA, catA1, qnrS1, OqxA, OqxB, sul1, tet(A), dfrA1, qacE | 395 |
| 1030839 | Urine | ant(2'')-Ia, ant(3'')-Ia, aac(6')-Ib-cr, *bla*OXA-1, *bla*OXA-48, *bla*SHV-158, *bla*TEM-1B, *bla*CTX-M-15, *bla*SHV-182, fosA, catA1, qnrS1, OqxA, OqxB, sul1, tet(A), dfrA1, qacE | 395 |
| 1030840 | Urine | ant(2'')-Ia, ant(3'')-Ia, rmtC, aac(6')-Ib-cr, *bla*OXA-1, *bla*OXA-48, *bla*TEM-1B, *bla*NDM-1, *bla*CTX-M-15, *bla*SHV-182, fosA, catA1, qnrS1, OqxA, OqxB, sul1, tet(A), dfrA1, qacE | 395 |
| 1030841 | Urine | aph(6)-Id, ant(2'')-Ia, aac(6')-Ib, ant(3'')-Ia, rmtC, aac(6')-Ib-cr, aph(3'')-Ib, *bla*OXA-1, *bla*OXA-48, *bla*SHV-158, *bla*TEM-1B, *bla*NDM-1, *bla*CTX-M-15, *bla*SHV-182, fosA, catA1, qnrB1, qnrS1, OqxA, OqxB, sul1, sul2, tet(A), dfrA14, dfrA1, qacE | 395 |
| 1030842 | Urine | aac(6')-Ib, armA, aac(6')-Ib-cr, aac(3)-IIa, *bla*OXA-1, *bla*OXA-48, *bla*SHV-110, *bla*TEM-1B, *bla*CTX-M-15, fosA, msr(E), mph(E), catA1, OqxA, OqxB | 377 |
| 1030844 | Urine | aac(6')-Ib, aph(3')-VIa, rmtC, aac(6')-Ib-cr, *bla*OXA-534, *bla*SHV-100, *bla*TEM-1B, *bla*CTX-M-15, fosA, fosA6, qnrS1, OqxA, OqxB, sul1, tet(A), dfrA14, dfrA1 | 14 |
| 1030845 | Urine | aac(6')-Ib, aph(3')-VIa, aac(6')-Ib-cr, *bla*OXA-1, *bla*OXA-48, *bla*TEM-1B, *bla*CTX-M-15, *bla*SHV-11, fosA, qnrS1, sul1, tet(A), dfrA1 | 395 |
| 1030846 | Urine | aac(6')-Ib-cr, aac(3)-IIa, *bla*OXA-1, *bla*OXA-48, *bla*SHV-158, *bla*TEM-1B, *bla*CTX-M-15, *bla*SHV-182, fosA, catA1, qnrS1, OqxA, OqxB, sul1, tet(A), dfrA1, qacE | 395 |
| 1030850 | Urine | ant(2'')-Ia, ant(3'')-Ia, rmtC, aac(6')-Ib-cr, *bla*OXA-1, *bla*OXA-48, *bla*TEM-1B, *bla*NDM-1, *bla*CTX-M-15, *bla*SHV-182, fosA, catA1, qnrS1, OqxA, OqxB, sul1, tet(A), dfrA1, qacE | 395 |
| 1030851 | Urine | aph(6)-Id, aac(6')-Ib, aac(6')-Ib-cr, aph(3'')-Ib, aac(3)-IIa, *bla*OXA-1, *bla*SHV-76, *bla*TEM-1B, *bla*CTX-M-15, fosA5, fosA, qnrB1, OqxA, OqxB, sul2, tet(A), dfrA14, qacE | 405 |
| 1030852 | Urine | armA, *bla*KPC-3, *bla*OXA-72, *bla*SHV-100, fosA5, fosA, msr(E), mph(E), OqxA, OqxB, qacE, OqxA, OqxB | 377 |
| 1030853 | Urine | armA, *bla*KPC-3, *bla*OXA-72, *bla*SHV-100, fosA5, fosA, , msr(E), mph(E), OqxA, OqxB, qacE | 101 |

Table S4.1 **ST groups of *K. pneumoniae* strains depending on their origin**

| **Hospital** | **Hospital department** | **No of isolates** | **ST** |
| --- | --- | --- | --- |
| MD003A | Intensive care unit | 2 | 395 |
| MD003A | Other departments | 4 | 23 **(K57)**, 395 |
| MD009A | Intensive care unit | 1 | 395 |
| MD013A | Intensive care unit | 6 | 11, 377, 395 |
|  | Internal medicine | 1 | 395 |
|  | Other departments | 2 | 395 |
| MD014A | Intensive care unit | 14 | 11, 147, 340, 377, 395 |
|  | Internal medicine | 5 | 101, 377, 395, 405 |
|  | Surgery | 2 | 23 **(K1)**, 101 |
|  | Urology | 6 | 14, 377, 395, 1037 |
|  | Other departments | 28 | 11, 15, **23 (K57)**, 101, 147, 340, 377, 380, 395 |
| MD015A | Intensive care unit | 9 | 25, 377, 395, 1026, 6381 |
|  | Urology | 4 | 395 |
| MD015B | Intensive care unit | 2 | 11, **23 (K57)** |
| MD016A | Intensive care unit | 1 | 395 |
|  | Other departments | 4 | 14, 37, 395 |
| MD017A | Paediatric intensive care unit | 2 | 1026 |
|  | Paediatry | 1 | 1026 |
| MD021A | Other departments | 1 | 395 |
| MD021D | Other departments | 1 | 340 |
| Ambulatory | Consultative | 3 | 23 (K57), 395 |

Table S4.2 **ST groups of *K. pneumoniae* strains depending on their origin**

| **ST** | **Number of isolates** | **% from total number of isolates** | **Hospital department** | **Hospital** |
| --- | --- | --- | --- | --- |
| 11 | 4 | 4,04 | Intensive care unit,  Other | MD013A, MD014A, MD15B, MD016A |
| 14 | 2 | 2,02 | Intensive care unit, Paediatry | MD016A |
| 15 | 2 | 2,02 | Internal medicine | MD014A, MD016A |
| **23** | 5 | 5,05 | Intensive care unit, Surgery, Urology | MD013A, MD014A, MD015A, MD015B,  Ambulatory |
| 25 | 1 | 1,01 | Intensive care unit | MD015A |
| 37 | 1 | 1,01 | Paediatry | MD016A |
| 101 | 3 | 3,03 | Intensive care unit, Urology | MD014A,  MD015A |
| 147 | 2 | 2,02 | Intensive care unit, Internal medicine | MD014A, MD21F |
| 340 | 3 | 3,03 | Intensive care unit, Urology,  Other departments | MD015A,  MD021D |
| 377 | 12 | 12,12 | Intensive care unit, Paediatric intensive care unit, Internal medicine | MD013A,  MD014A,  MD015A,  MD016A,  MD017A |
| 380 | 1 | 1,01 | Surgery | MD013A |
| 395 | 56 | 56,56 | Intensive care unit, Internal medicine, Urology, Gynecology,  Other departments | MD013A, MD014A,  MD015A,  MD016A, MD021A, Ambulatory |
| 405 | 1 | 1,01 | Intensive care unit | MD015B |
| 1026 | 4 | 4,04 | Intensive care unit, Paediatric intensive care unit,  Paediatry | MD015A,  MD017A |
| 1037 | 1 | 1,01 | Internal medicine | MD015B |
| 6381 | 1 | 1,01 | Intensive care unit | MD015A |

Table S5. **Hypervirulent *Klebsiella pneumoniae* depending on ST**

| **No isolate** | **ST** | **Clonal lineage** | **Virulence score** | **Department** | **Institution** | **Isolation year** |
| --- | --- | --- | --- | --- | --- | --- |
| 1030835 | ST23 | K1 | 5 | Surgical | MD014A | 2023 |
| 1030713 |  | K57 | 4 | Intensive care unit | MD014A | 2020 |
| 1030777 |  |  | 4 | Outpatient | Ambulatory | 2020 |
| 1030744 |  |  | 4 | Other | MD014A | 2023 |
| 1030766 | ST25 | K39 | 4 | Intensive care unit | MD014A | 2022 |
| 1030830 | ST380 | K2 | 5 | Other | MD014A | 2023 |
| 1030710 | ST395 | K39 | 4 | Intensive care unit | MD015A | 2020 |
| 1030711 |  |  | 4 | Intensive care unit | MD015A | 2020 |
| 1030722 |  |  | 4 | Other | MD014A | 2021 |
| 1030723 |  |  | 4 | Other | MD014A | 2021 |
| 1030728 |  |  | 4 | Other | MD015A | 2021 |
| 1030736 |  |  | 4 | Other | MD014A | 2022 |
| 1030750 |  |  | 4 | Other | MD013A | 2022 |
| 1030753 |  |  | 4 | Intensive care unit | MD015A | 2022 |
| 1030757 |  |  | 4 | Intensive care unit | MD013A | 2022 |
| 1030759 |  |  | 4 | Intensive care unit | MD013A | 2022 |
| 1030767 |  |  | 4 | Intensive care unit | MD016A | 2022 |
| 1030780 |  |  | 4 | Intensive care unit | MD003A | 2023 |
| 1030784 |  |  | 4 | Other | MD003A | 2023 |
| 1030785 |  |  | 4 | Intensive care unit | MD003A | 2023 |
| 1030805 |  |  | 4 | Intensive care unit | MD014A | 2023 |
| 1030810 |  |  | 4 | Intensive care unit | MD014A | 2023 |
| 1030812 |  |  | 4 | Other | MD014A | 2023 |
| 1030819 |  |  | 4 | Intensive care unit | MD014A | 2023 |
| 1030828 |  |  | 4 | Urology | MD014A | 2023 |
| 1030831 |  |  | 4 | Other | MD014A | 2023 |
| 1030836 |  |  | 4 | Intensive care unit | MD014A | 2023 |
| 1030837 |  |  | 4 | Other | MD013A | 2023 |
| 1030838 |  |  | 4 | Other | MD013A | 2023 |
| 1030839 |  |  | 4 | Other | MD013A | 2023 |
| 1030840 |  |  | 4 | Urology | MD016A | 2023 |
| 1030841 |  |  | 4 | Urology | MD013A | 2023 |
| 1030850 |  |  | 4 | Other | MD016A | 2023 |

Table S6. **Resistance genes harboured by ST23 K57 lineage isolates**

| **Isolate ID** | **Lineage** | **Resistance genes** | | | | | | | | | |
| --- | --- | --- | --- | --- | --- | --- | --- | --- | --- | --- | --- |
| **1030713** | **K57** | *bla*SHV-51 | *bla*CTX-M-55 | *bla*LAP-2 | *bla*SHV-33 | *bla*SHV-45 | - |  | - | - | *bla*OXA-1 |
| **1030777** | **K57** | *bla*SHV-51 | *bla*CTX-M-55 | *bla*LAP-2 | - | *bla*SHV-45 |  | *bla*OXA-72 | - | - | *bla*OXA-1 |
| **1030744** | **K57** | *bla*SHV-51 | *bla*CTX-M-55 | *bla*LAP-2 | - | - | - |  | *bla*OXA-48 | *bla*TEM-1B | *bla*OXA-1 |
| **1030783** | **K57** | *bla*SHV-51 | *bla*CTX-M-55 | *bla*LAP-2 | *bla*SHV-33 | *bla*SHV-45 | - | - |  | - | *bla*OXA-1 |
| **1030835** | **K1** | - | - | - | - | - | *bla*SHV-190 | - | - | - | - |
